# Prolonged immune activation in post-acute sequelae of SARS-CoV-2: neutrophil dynamics and therapeutic insights

**DOI:** 10.1038/s12276-025-01539-5

**Published:** 2025-09-24

**Authors:** Mina Yu, Suhee Hwang, Hobin Jang, Dongbin Park, Jaemoo Kim, Juryeon Gil, Jeong Ho Choi, Seung-Gyu Jang, Isaac Choi, Yuri Jung, Woohyun Kwon, Se-Mi Kim, Young-Il Kim, Hyunjoon Kim, Taehwan Oh, Joo Sang Lee, Min-Suk Song, SangJoon Lee, Young Ki Choi

**Affiliations:** 1 Center for Study of Emerging and Re-emerging Viruses, Korea Virus Research Institute, Institute for Basic Science, Daejeon, Republic of Korea; 2 College of Medicine and Medical Research Institute, Chungbuk National University, Cheongju, Republic of Korea; 3 Department of Metabiohealth, Sungkyun Convergence Institute, Sungkyunkwan University, Suwon, Republic of Korea; 4 Virus Research Resource Center, Korea Virus Research Institute, Institute for Basic Science, Daejeon, Republic of Korea; 5 Department of Biological Science, Ulsan National Institute of Science and Technology, Ulsan, Republic of Korea

**Keywords:** Viral infection, Infection

## Abstract

Post-acute sequelae of SARS-CoV-2 (PASC) is characterized by persistent symptoms such as fatigue, respiratory complications and cognitive dysfunction, affecting approximately 13.5% of SARS-CoV-2-infected individuals. Despite its clinical significance, the mechanisms driving PASC remain poorly understood. Here, to address this, we utilized a *Phodopus roborovskii* hamster model to investigate the long-term effects of SARS-CoV-2 infection compared with influenza A virus. While 46.25–47.50% of hamsters survived SARS-CoV-2 or influenza A virus H1N1 infection, 13.75% of SARS-CoV-2 survivors exhibited impaired weight recovery, severe lung pathology and significant neutrophil accumulation, defining the PASC group. Single-cell RNA sequencing of bronchoalveolar lavage fluid, lung and spleen at 30 days post-infection revealed hallmark PASC gene signatures uniquely upregulated in the PASC group. This was accompanied by elevated neutrophil levels and reduced macrophage populations, indicative of disrupted myeloid cell differentiation. Immunohistochemistry further detected persistent SARS-CoV-2 S1 subunit antigen in the lungs of PASC hamsters at 30 days post-infection, coinciding with marked neutrophil infiltration, which probably drove prolonged inflammatory responses. Indeed, the neutrophils in the PASC group exhibited sustained upregulation of inflammation-related genes, including *FPR2*, *MMP9* and *S100A9*, which are associated with neutrophil degranulation and extracellular trap formation. Importantly, targeting neutrophil-mediated inflammation with small-molecule inhibitors substantially reduced PASC phenotypes. Among these, Sivelestat, a neutrophil elastase inhibitor, demonstrated the most pronounced efficacy, reducing PASC incidence and mortality, and markedly reducing neutrophil levels. These findings underscore the critical role of neutrophil activation in driving lung damage and chronic inflammation during PASC, offering promising therapeutic strategies for mitigating long-term COVID-19 sequelae.

## Neutrophil activation drives long-term COVID-19 lung damage

The COVID-19 pandemic, caused by the SARS-CoV-2 virus, has had a major impact on global health. While vaccines have helped reduce severe cases, new variants and long-term effects, known as long COVID, remain concerns. Long COVID includes symptoms such as fatigue and breathing issues that persist after recovery. This study explores these long-term effects using a hamster model that can induce long COVID symptoms, as observed in patients with COVID-19. Researchers infected hamsters with the virus and studied their tissues using advanced techniques such as single-cell RNA sequencing and histopathological examination. They found that certain immune cells, especially neutrophils, were overly active in long COVID cases. The study also tested drugs targeting these cells and found that Sivelestat, a neutrophil elastase inhibitor, reduced symptoms and mortality. This suggests that targeting neutrophil activity could help manage long COVID.

This summary was initially drafted using artificial intelligence, then revised and fact-checked by the author.

## Introduction

The coronavirus disease 2019 (COVID-19) pandemic, caused by severe acute respiratory syndrome coronavirus 2 (SARS-CoV-2), has profoundly impacted global health and economies since its emergence in late 2019^1,2^. Although vaccination efforts have substantially reduced the severity and mortality rates associated with SARS-CoV-2 infection^3^, the emergence of variants continues to challenge vaccine efficacy^4^, necessitating ongoing vigilance and adaptable public health strategies. Moreover, beyond the acute phase, the long-term consequences of SARS-CoV-2 infection, collectively termed post-acute sequelae of SARS-CoV-2 (PASC) or long COVID, have emerged as a pressing global health concern^5,6^.

PASC is characterized by persistent symptoms that can last for weeks or months after the initial infection^7^. These symptoms include fatigue, respiratory issues, cognitive dysfunction and a general decline in quality of life. The complexity and variability of PASC symptoms have made it challenging to develop standardized treatments, leaving many patients without effective management options. With the growing population of COVID-19 survivors, the burden of PASC is expected to escalate, underscoring the urgent need for mechanistic research and targeted interventions. Despite substantial progress, most PASC studies rely on clinical data from minimally invasive samples, such as peripheral blood mononuclear cells or bronchoalveolar lavage fluid (BALF)^8,9^. These studies often lack multitissue analysis and are limited by inconsistent timing for symptom assessments, complicating cross-study comparisons and slowing the development of effective therapies. Thus, a more comprehensive, multitissue approach, incorporating single-cell resolution and standardized analysis time points, is essential to uncover the cellular and molecular pathways driving PASC.

In this study, we addressed these challenges using *Phodopus roborovskii* (*P. roborovskii*) hamsters, an established model that closely mimics the clinical manifestations of SARS-CoV-2 infection in humans, including severe disease progression and a measurable case fatality rate^10^. This model offers a unique balance of practicality and relevance, making it an invaluable tool for investigating long COVID. Using this model, we performed an in-depth analysis of the long-term effects of SARS-CoV-2 infection and compared them with those of influenza A virus (IAV). Through multitissue single-cell RNA sequencing (scRNA-seq) and histopathological examinations, we identified specific disruptions in myeloid cell populations and their differentiation pathways following SARS-CoV-2 infection, providing critical insights into PASC mechanisms. Notably, we observed a prolonged accumulation of the SARS-CoV-2 spike protein S1 subunit (S1) antigen in the lungs up to 30 days post-infection (dpi), accompanied by significant neutrophil infiltration in the PASC group. Furthermore, targeting neutrophil-mediated inflammatory pathways with Sivelestat, a neutrophil elastase inhibitor, markedly reduced PASC incidence and mortality by over 50%. These findings highlight neutrophil activation as a central driver of PASC pathogenesis and underscore the therapeutic potential of targeting neutrophils to mitigate long COVID.

## Materials and methods

### Experimental animals and ethics statement

The IAV and SARS-CoV-2 negative *P. roborovskii* hamsters (6–8 weeks old) were purchased from Dooyeol Biotech. Animal studies were conducted following experimental procedures approved by the Institutional Animal Care and Use Committee of Chungbuk National University (approval number CBNUA-194R-22-01) and the Institute for Basic Science (approval number IBS-2024-001). All experiments were conducted in biosafety level 3 laboratories at Chungbuk National University (KCDC-14-3-07) and the Institute for Basic Science (KCDC-23-3-06).

### Virus infection and chemical administration

A mouse-adapted pandemic H1N1 IAV (maCA04/09)^11^ and a SARS-CoV-2 delta variant strain (GISAID accession number EPI_ISL_10189547) isolated from a patient with confirmed COVID-19 in South Korea were cultured in MDCK (Madin-Darby Canine Kidney cells) and Vero E6 (African green monkey kidney cells) cells, respectively^12,13^. For virus infections, groups of hamsters were anesthetized using isoflurane, and administered 30 µl of SARS-CoV-2 at a titer of 10^5.5^ tissue culture infective dose 50% (TCID_50_)/ml or IAV at 10^7.0^ TCID_50_/ml through intranasal routes. Body weight and survival were monitored every 2 dpi until the end of experiments. Animals that experienced more than a 25% reduction in body weight were humanely euthanized.

WRW4 (Selleckchem) was used as an FPR2 antagonist, Paquinimod (TargetMol) as an S100A8/A9 inhibitor and Sivelestat (MedChemExpress) as a neutrophil elastase inhibitor. All compounds were dissolved in 2% dimethyl sulfoxide (DMSO) in saline. Drug administration was performed using two schedules: early treatment from 5 to 11 dpi (*n* = 50) and delayed treatment from 15 to 21 dpi (*n* = 20) following SARS-CoV-2 infection. With the exception of the negative control and nontreated groups, the following treatments were applied: 2% DMSO in saline (50 μl, intraperitoneally), Paquinimod (10 mg/kg/day, 50 μl, orally) and Sivelestat (10 mg/kg/day, 50 μl, intraperitoneally), administered daily for 7 days. WRW4 (8 mg/kg/day, 50 μl, intraperitoneally) was administered by 2 days for a total of 4 days.

### Viral titer determination

For titration of TCID_50_, tissue homogenates were prepared by homogenizing entire tissue samples in 0.7 ml of virus culture media containing 2% penicillin–streptomycin (P/S, Gibco) using a TissuLyser II (Qiagen). The homogenates were clarified through three rounds of centrifugation at 12,000 rpm for 10 min. Influenza virus titers were determined by infecting MDCK cells with 10-fold serial dilutions of tissue homogenates or BALF in Eagle’s minimum essential medium (Gibco) containing 1% P/S, followed by incubation with Eagle’s minimum essential medium supplemented with 1 μg/ml TPCK-trypsin (Worthington Biochemical) at 37 °C with 5% CO_2_ for 72 h. Similarly, SARS-CoV-2 titers were assessed by infecting Vero E6 cells with 10-fold serial dilutions of tissue homogenates or BALF in Dulbecco’s modified Eagle medium (Gibco) with 1% P/S, followed by incubation with Dulbecco’s modified Eagle medium containing 2% fetal bovine serum and 1% P/S at 37°C with 5% CO_2_ for 72 h (ref. ^12^). Cytopathic effects were monitored daily, and viral titers were calculated as log_10_ TCID_50_/ml using the Reed–Muench method^14^.

### RNA extraction and quantitative real-time PCR (qRT–PCR)

Harvested hamster tissues were placed in TRIzol (Invitrogen) and homogenized using an Omni Tissue Homogenizer (OMNI International). RNA was extracted from the homogenized tissues following the TRIzol RNA extraction protocol. The extracted RNA was then used to synthesize complementary DNA (cDNA) with SuperScript III Reverse Transcriptase (Invitrogen). For the synthesis, oligo d(T) and random primers (Promega) were used. qRT–PCR was conducted using the synthesized cDNA, IQ SYBR Green SuperMix (Bio-Rad) and primers (Supplementary Table 1) and was detected by CFX Opus 96 (Bio-Rad).

### Enzyme-linked immunosorbent assay (ELISA)

The GM-CSF and G-CSF ELISAs were performed using the Hamster GM-CSF Antibody ELISA Kit (MyBioSource) and G-CSF ELISA Kit (MyBioSource), following the protocols provided by the manufacturer.

### Histopathological assay and RNAscope

Whole-lung samples from mock and virus-infected mice were collected at 5, 15 and 30 dpi. The samples were fixed in 10% neutral-buffered formalin, embedded in paraffin and stained with hematoxylin and eosin (H&E) or Masson’s trichrome (MT) staining solution. Immunohistochemistry (IHC) was performed using the Leica Bond Rx Research Stainer (Leica Microsystems) with bond polymer refine detection. For the detection of SARS-CoV-2 antigens, tissue sections were stained with anti-SARS-CoV-2 spike protein S1 antibody (HL134, Abcam) and anti-SARS-CoV-2 nucleocapsid rabbit monoclonal antibody (EPR24334-118, Abcam). Detection was performed using peroxidase AffiniPure goat anti-rabbit IgG (H + L) secondary antibody (Jackson ImmunoResearch). For detection of IAV antigens, sections were stained with an anti-nucleoprotein antibody (H16-L10-4R5, GeneTex), followed by Peroxidase AffiniPure Goat Anti-Mouse IgG (H + L) secondary antibody (Jackson ImmunoResearch).

The RNAscope in situ hybridization assay used V-Influenza-H1N1-H5N1-M, V-nCoV2019-S probes (ACD Bio) and the RNAscope 2.5 HD Detection Kit – RED (ACD Bio), following the manufacturer’s protocol. All the stained sections were visualized using an Axio scan Z1 (Carl Zeiss), and fibrosis areas were analyzed with pixel classification in QuPath software (v0.5.0).

### Multiplex immunofluorescence

The samples processed using the Leica Bond Rx Research stainer (Leica Microsystems) with Opal 3-plex detection kit (PerkinElmer). Anti-myeloperoxidase (MPO) antibody (Abcam) and anti-CD68 antibody (Abcam) were used for detection, followed by peroxidase-conjugated anti-rabbit antibody (Jackson Immuno Research Laboratories). Fluorophore labeling was performed using Opal 570 and 690 fluorophores, and the samples were mounted with Prolong Gold Antifade reagent with DAPI (Invitrogen). Quantitative analysis was conducted using QuPath software (v0.5.0), performing object classification to quantify results.

### Library preparation

The single-cell RNA libraries were prepared utilizing the 10x Genomics Chromium Single Cell 3′ Reagent Kit (v3.1, 10x Genomics) based on the manufacturer’s instructions. In brief, from each tissue sample, approximately 8,000 cells were loaded in one channel. In each droplet, cDNA was generated through reverse transcription. The quality of the resulting cDNA libraries was evaluated using the 4150 TapeStation 4150 (Agilent) and quantified with the High Sensitivity D1000 Screen Tape (Agilent) based on the manufacturer’s library quantification protocol. Following cluster amplification of denatured templates, sequencing was performed in a paired-end fashion (2 × 100 bp) using the Illumina Novaseq 6000 platform (Illumina).

### Quality control and computational analyses of scRNA-seq data

Genomes of SARS-CoV-2 delta variant, H1N1 IAV and *P. roborovskii* hamsters (NCBI accession number GCF_943737965.1) were retrieved from the NCBI database (as of October 2023). To improve the gene annotation, we used the Eggnog-mapper software (v2.1.11)^15^. The scRNA-seq data were processed using Cell Ranger from 10x Genomics (v7.2.0; www.10xgenomics.com) and analyzed with the Seurat package (v4.1.1)^16^. In brief, following the alignment of sequencing reads^17^, low-quality cells were removed on the basis of the unique molecular identifier counts (<400) and gene counts (<200 or >8,000), and the proportion of unique molecular identifier counts mapped to mitochondrial gene/cell (≥20%). In addition, cell-free RNA and doublets were removed using SoupX (v1.6.2) and DoubletFinder (v2.0), respectively^18,19^. Qualified scRNA-seq data were then normalized using SCTransform^20^, reduced in dimensionality by principal component analysis and integrated using reciprocal principal component analysis. The batch-corrected matrices were constructed using the *k*-nearest neighbor graph with the *FindNeighbors* function (*k* = 50). Finally, single-cell transcriptomic networks for three tissue types were visualized using the Uniform Manifold Approximation and Projection (UMAP) method. Clustering was performed using the Louvain algorithm, and cell types were annotated on the basis of canonical differentially expressed genes (DEGs, avg_log_2_fold change (FC) >0.25; *P* < 0.05, Benjamini–Hochberg corrected) and verified with CellKb (v2.6; https://www.cellkb.com/). For trajectory analyses of myeloid cell populations, we used the Monocle 3 package (v1.3.5)^21^.

### Composition changes in cell populations

From the scRNA-seq data, the cell type percentages were calculated by dividing the number of cells in each type by the total cell count in the control and diseased groups. To minimize bias from small sample sizes, population changes were quantified by dividing diseased cell counts by controls for all hamster pairs. Following logarithmic formation (log_2_), we conducted statistical analyses on the relative variation in cell count between the control and diseased groups using a Wilcoxon rank-sum test.

### Scoring cells for biological functions

For the functions of interest in selected cell types, we assessed the combined expressions of a set (module) of relevant genes per cell using the AddModuleScore in the Seurat (v4.1.1)^16^. To this end, the Gene Ontology Biological Process (GOBP) database, along with the 50 hallmark gene sets and WikiPathway gene sets from the Molecular Signatures Database (MSigDB, v7.5.1), were used^22^. Module scores for neutrophil granule production and aging were evaluated on the basis of gene sets informed by prior studies on neutrophil function and aging processes^23,24^. We used the Wilcoxon rank-sum test to statistically compare variations in all these module scores between diseased versus control groups, respectively.

### Cell-to-cell communication analysis

Cell-to-cell communication networks were performed using CellChat R package (v2.1.2)^25^. The lung scRNA-seq dataset was input into the *createCellChat* function, followed by preprocessing with the *identifyOverExpressedGenes* and *identifyOverExpressedInteractions* functions. Communication probabilities were computed using the *computeCommunProb* function with type = truncatedMean and trim = 0.2 and inferred at signaling pathway levels by the *computeCommunProbPathway* function. The TGF-β signaling pathway was visualized by the *netVisual_aggregate* function.

### Functional analyses of group-specific upregulated and downregulated gene sets

To identify sets of genes that are specifically upregulated or downregulated in the diseased groups, we calculated the FC in the expression of genes and statistical significance (Wilcoxon rank-sum test) in the respective diseased groups compared with the control counterparts. From these comparisons, the group-specific upregulated genes were considered as those that showed the log_2_FC >0.25 and *P* value <0.05 in each diseased group but not in the other two. Conversely, group-specific downregulated genes were defined as those showing the log_2_FC <0.25 and *P* value <0.05 in the given group but not in the others. Functional analyses using these group-specific upregulated or downregulated genes were performed by EnrichR (v3.2) software with the databases of GOBP, MSigDB Hallmark, Kyoto Encyclopedia of Genes and Genomes (KEGG) and/or Reactome^26^. Gene pathway analysis was conducted with Ingenuity pathway analysis (IPA; Qiagen).

### Flow cytometry

To assess the composition of immune cell populations, lung and spleen tissues were collected from euthanized animals at 5, 15 and 30 dpi. Tissues were dissociated into single-cell suspensions using the gentleMACS Octo Dissociator (Miltenyi Biotec) and filtered through a 70-μm cell strainer. Approximately 1 × 10⁶ cells per sample were incubated with anti-mouse CD16/CD32 antibody (Mouse Fc Block, BD Biosciences) at 4 °C for 30 min to block Fc receptors. After centrifugation and removal of the supernatant, cells were stained at 4 °C for 30 min with the following surface antibodies: BV421-conjugated anti-human CD11b antibody (clone ICRF44, BD Biosciences), PerCP-Cy5.5-conjugated anti-human CD14 antibody (clone M5E2, BD Biosciences) and FITC-conjugated anti-mouse Ly-6G antibody (clone 1A8, BioLegend). Cells were then washed with Flow Cytometry Staining Buffer (Invitrogen) and fixed in 4% paraformaldehyde.

For intracellular staining, fixed cells were permeabilized using the Cytofix/Cytoperm Plus Fixation/Permeabilization Solution Kit (BD Biosciences) at 4 °C for 30 min. After washing twice with Perm/Wash buffer, cells were incubated with PE-conjugated anti-human, mouse, ferret CD79a antibody (clone HM47, Invitrogen) and Alexa Fluor 647-conjugated anti-human CD3 antibody (clone CD3-12, Bio-Rad) at 4 °C for 30 min. Following two final washes, cells were fixed again in 4% paraformaldehyde and analyzed on a FACSymphony A3 Cell Analyzer (BD Biosciences). Flow cytometric data were processed using FlowJo software (v10.9.0).

### Quantification and statistical analysis

Data are shown as mean ± standard deviations (s.d.). The statistical analyses of in vitro and in vivo studies were performed using one-way analysis of variance (ANOVA) (Figs. 1, 3 and 5–7 and Supplementary Figs. 1, 6 and 7). For other analyses, the Wilcoxon rank-sum (Mann–Whitney *U*) test was used to assess statistical significance (Figs. 2–5 and 8 and Supplementary Figs. 2–5). Statistical analyses were performed using GraphPad Prism 10 (v10.3.1). Detailed statistical methods and results are provided in each figure legend.

## Results

### Long-term weight loss and lung pathology in *P. roborovskii* hamsters after SARS-CoV-2 infection

**Fig. 1:**
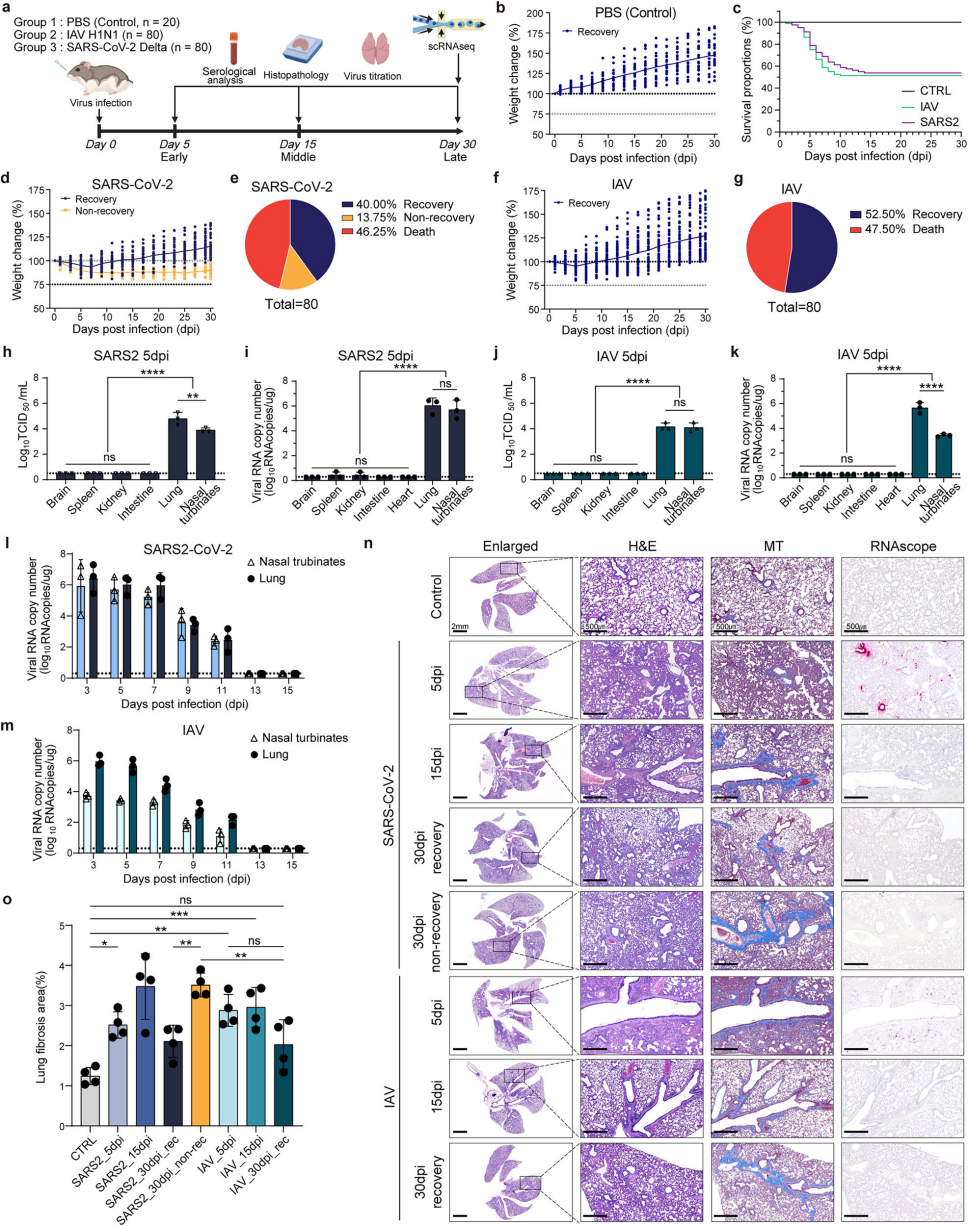
Experimental design and analysis of body weight changes, viral load and tissue pathology in SARS-CoV-2 and IAV infection. **a** A schematic of the experiment and sample collection. Samples from each group were collected at 5, 15 and 30 dpi for serological analysis, histopathology and virus titration. Notably, tissue samples at 30 dpi were prepared for scRNA-seq analysis. Created with BioRender.com. **b** Body weight change data of the control group (CTRL, *n* = 20). **c** Survival rate of all three groups. **d** Body weight change data of the SARS-CoV-2 infection group (*n* = 80). **e** Proportions of the SARS-CoV-2 infection group based on body weight changes and mortality at 30 dpi. **f** Body weight change data of the IAV infection group (*n* = 80). **g** Proportions of the IAV infection group based on body weight changes and mortality at 30 dpi. **h**, **i** TCID_50_ (**h**) and viral RNA copy number (**i**) of various tissues in the SARS-CoV-2 infection group at 5 dpi (*n* = 3). **j**, **k** TCID_50_ (**j**) and viral RNA copy number (**k**) of various tissues in the IAV infection group at 5 dpi (*n* = 3). **l**, **m** Viral RNA copy number in nasal turbinates and lung tissues at 2-day intervals post-infection for SARS-CoV-2 (**l**) and IAV (**m**) (*n* = 3). **n** H&E, MT staining and RNAscope images. Enlarged images (scale bars, 2 mm) show whole lung images of H&E, with high-magnification images of corresponding areas stained with H&E, MT and RNAscope (scale bars, 500 μm). Light blue in MT-stained images indicates fibrosis, and red dots in RNAscope images represent detected viral RNA. **o** Graph showing the percentage of fibrosis area relative to total lung area based on MT staining results for each group (*n* = 4). Data for all graphs are presented as means ± s.d. Statistical significance is indicated as follows: *P < 0.05, **P < 0.01, ***P < 0.001, ****P < 0.0001, ns (*P* > 0.05), one-way ANOVA. SARS2, SARS-CoV-2.

To investigate the long-term effects of SARS-CoV-2 infection and determine whether these effects are specific to SARS-CoV-2 infection, we conducted a comparative longitudinal study using *P. roborovskii* hamster^10^. A group of hamsters (*n* = 80) was infected with either the SARS-CoV-2 Delta variant (10^5.5^ TCID_50_/ml, 1 lethal dose, 50% (LD_50_)) or IAV H1N1 (10^7.0^ TCID_50_/ml, 1 LD_50_), while a control group received phosphate-buffered saline (CTRL) (Fig. 1a). Weight changes were monitored for 30 dpi, with animals euthanized at 5, 15 and 30 dpi for serological, histopathological and viral load analyses. The phosphate-buffered saline-treated control group gained weight throughout the study (Fig. 1b). By contrast, the SARS-CoV-2-infected group showed marked weight loss by 7 dpi, with a mortality rate of 46.25% within the first 2 weeks (Fig. 1c–e). Among the SARS-CoV-2-infected group, 40% of the survivors regained their preinfection body weight by 30 dpi (SARS-CoV-2_recovery group), whereas 13.75% failed to recover and were classified as non-recovery, showing persistent weight loss (SARS-CoV-2_non-recovery group) (Fig. 1d, e). By comparison, the IAV-infected group showed a similar mortality rate of 47.50% by 10 dpi but demonstrated full weight recovery in all survivors by 30 dpi (IAV_recovery group) (Fig. 1c, f, g).

To investigate whether the observed differences in recovery dynamics were related to variations in viral titers and replication kinetics, respiratory tissues were collected at 5, 15 and 30 dpi. In the SARS-CoV-2 group, infectious virus was recovered at 5 dpi, with lung titers approximately 10 times higher than those in the nasal turbinates, despite similar viral RNA copy numbers in both tissues (Fig. 1h, i). Similarly, in the IAV group, both infectious virus and viral RNA were detected in nasal turbinates and lung tissues at 5 dpi (Fig. 1j, k). However, by 15 and 30 dpi, no infectious virus or viral RNA was detectable in either group (Supplementary Fig. 1a–h). Viral RNA levels in nasal turbinates and lungs were analyzed at two-day intervals from 3 to 15 dpi, revealing higher viral titers in the lungs, peaking at 3 dpi and declining until undetectable by 13 dpi (Fig. 1l, m and Supplementary Fig. 1a–h). These results indicate that the persistent weight loss in the SARS-CoV-2 group is unrelated to ongoing viral replication.

Histopathological examination revealed that virus-induced lung lesions, including fibrosis, persisted until 30 dpi only in the SARS-CoV-2_non-recovery group (Fig. 1n, o). By contrast, the IAV_recovery and SARS-CoV-2_recovery groups showed significant improvement in lung pathology by 30 dpi, as confirmed by H&E and MT staining. The IAV_recovery group showed fibrosis at 5 dpi, which resolved over time, while fibrosis persisted in the SARS-CoV-2_non-recovery group (Fig. 1n, o). RNA in situ hybridization (RNAscope) confirmed viral RNA presence only until 5 dpi in both groups, consistent with TCID_50_ and qRT–PCR results (Fig. 1h–k, n and Supplementary Fig. 1a–h). These findings suggest that persistent weight loss and severe lung pathology in the SARS-CoV-2_non-recovery group are driven by sustained lung damage rather than prolonged viral replication, highlighting a distinct pathophysiological feature of long COVID in this animal model^27^.

### Transcriptional profiling of SARS-CoV-2 non-recovery group reveals hallmark PASC genes

**Fig. 2:**
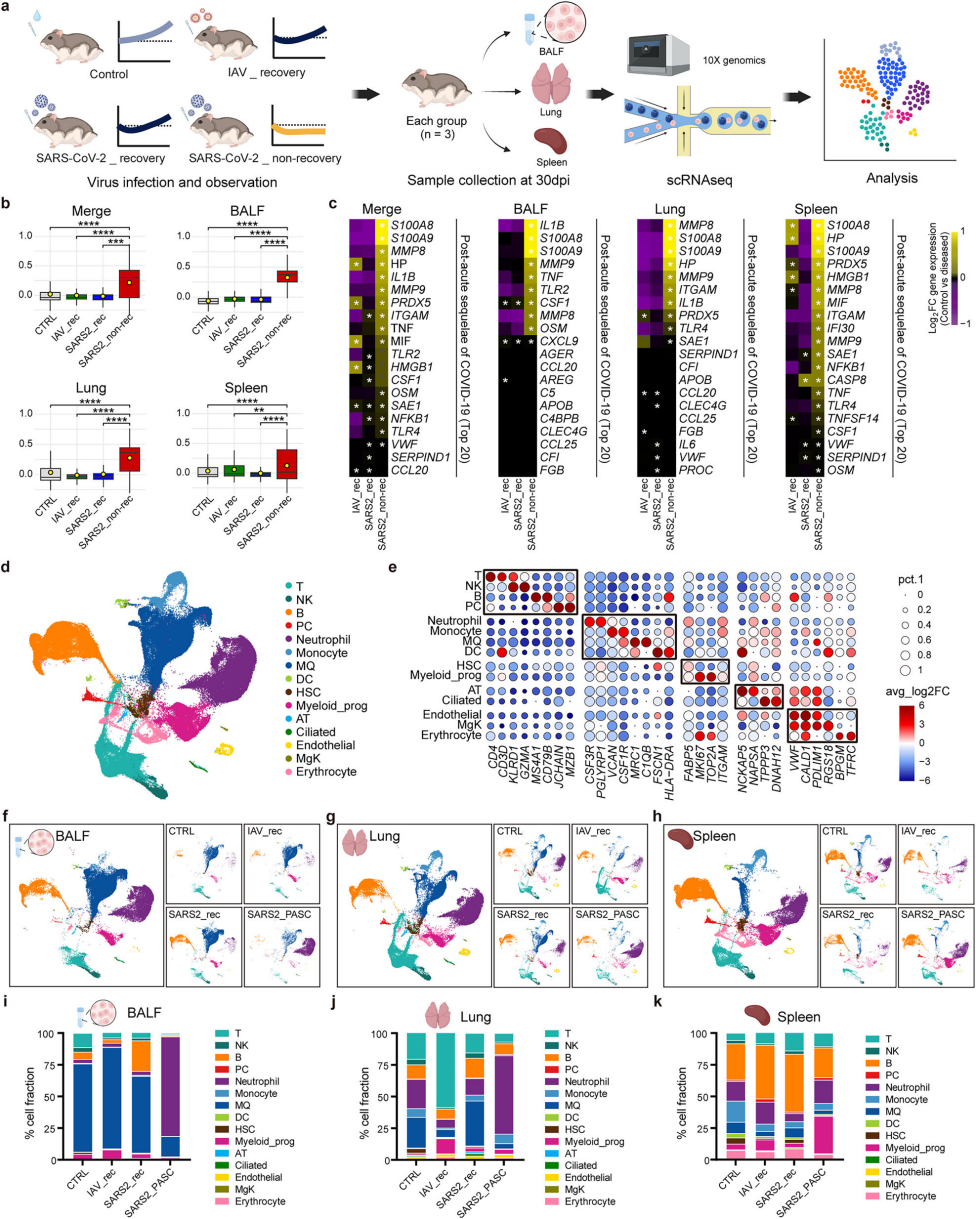
Analysis of hallmark PASC gene expressions and cell proportions based on scRNA-seq data. **a** A schematic overview of the scRNA-seq workflow from sample preparation to analysis. Created with BioRender.com. **b** Module scores calculated from the expression value of human PASC marker genes across the merged tissue (BALF, lung and spleen combined) as well as individual BALF, lung and spleen tissues. The boxes display the interquartile range (IQR = Q3–Q1; the 25th (Q1) to the 75th percentiles (Q3)), with the centerline denoting the median and the yellow dot representing the mean. **c** Heatmaps depicting the expression of representative human PASC hallmark genes in the merged tissue and individual tissues. The top 20 genes are only described, based on the log_2_ fold-change (log_2_FC) of their gene expression compared to the control. **d** UMAP plot showing the colored visualization of 15 cell types generated from scRNA-seq analysis of BALF, lung and spleen tissues. **e** Dot plot annotation of the 15 distinct cell types based on 30 marker genes. Dot size and color indicate the percentage (pct.1) of cells expressing each marker and the average log_2_ fold-change (avg_log_2_FC) of marker expression, respectively. **f**–**h** UMAP plots depicting the distribution of cell populations in BALF (**f**), lung (**g**) and spleen (**h**) tissues. Small insets illustrate group-specific distributions within each tissue. **i**–**k** Proportions of each cell type in control and diseased groups, identified in BALF (**i**), lung (**j**) and spleen (**k**) tissues. Some significance was determined using the Wilcoxon rank-sum test, and all *P* < 0.05 are represented with an asterisk owing to space limitations (**c**). Other statistical significances are indicated as follows: *P < 0.05, **P < 0.01, ***P < 0.001, ****P < 0.0001, ns (*P* > 0.05). The *P* values were estimated using Wilcoxon rank-sum test. CTRL, control group; IAV_rec, IAV_recovery group; SARS2_rec, SARS-CoV-2_recovery group; SARS2_PASC, SARS-CoV-2_non-recovery group; T, T cells; NK, natural killer cells; B, B cells; PC, plasma cells; DC, dendritic cells; HSC, hematopoietic stem cells; Myeloid_prog, myeloid progenitor cells; AT, pulmonary alveolar type I and type II cells; Ciliated, ciliated cells; Endothelial, endothelial cells; MgK, megakaryocytes.

To elucidate the pathophysiological differences among the IAV_recovery, SARS-CoV-2_recovery and SARS-CoV-2_non-recovery groups, we performed scRNA-seq with the BALF, lung and spleen collected at 30 dpi, a stage where distinct group-specific features are evident (Fig. 2a). At first, we investigated whether the SARS-CoV-2_non-recovery group exhibited characteristics similar to human cases of PASC. Interestingly, the SARS-CoV-2_non-recovery group showed significant upregulation of hallmark PASC-associated genes^28–31^, with over 65.00% (26/40) of these genes elevated across all three tissues, including *S100A8*, *S100A9*, *MMP8*, *IL1B*, *TNF* and others (Fig. 2b, c and Supplementary Fig. 2a). This transcriptional signature, absent in the recovery groups (‘SARS2_rec’ and ‘IAV_rec’), aligns with human PASC profiles, validating the SARS-CoV-2_non-recovery group as a model for PASC. Consequently, we refer to this group as ‘SARS2_PASC,’ while the remaining groups are designated as ‘CTRL’, ‘SARS2_rec’ and ‘IAV_rec’ for simplicity.

Next, we conducted scRNA-seq analysis of 158,116 individual cell transcriptomes from 36 tissue samples, including lung, BALF and spleen (Supplementary Table 2). Unsupervised clustering and differential gene expression analysis identified 15 major cell types, including immune, progenitor, epithelial and endothelial cells (Figs. 2d–h and Supplementary Table 3). Cell-type quantification revealed that neutrophil and macrophage (MQ) compositions in both recovery groups and CTRL were comparable across BALF and lung tissues (neutrophils 3.04–23.08%, MQ 6.12–80.05%) (Fig. 2i, j, Supplementary Fig. 2b, c and Supplementary Table 4). In stark contrast, the SARS2_PASC group exhibited a marked increase in neutrophils (61.88–78.45%) and a decrease in MQ (4.16–16.02%) in both tissues. In addition, myeloid progenitor cells notably accumulated in the spleen of the SARS2_PASC group, whereas they were scarce in the recovery groups (Fig. 2k, Supplementary Fig. 2d and Supplementary Table 4).

Beyond these PASC-specific alterations, recovery groups exhibited distinct immune cell dynamics. For example, the SARS2_rec group displayed increased B cell proportions in BALF and spleen compared with CTRL and other disease groups (Fig. 2i, k). Conversely, the IAV_rec group showed a twofold increase in T cell composition in the lung relative to CTRL, a change absent in the SARS2_PASC and SARS2_rec groups (Fig. 2j). These findings highlight unique transcriptional profiles and cellular changes linked to SARS-CoV-2 and IAV infections, emphasizing the distinct immune dysregulation associated with SARS2_PASC.

### Sustained neutrophil differentiation and accumulation in the SARS2_PASC group

**Fig. 3:**
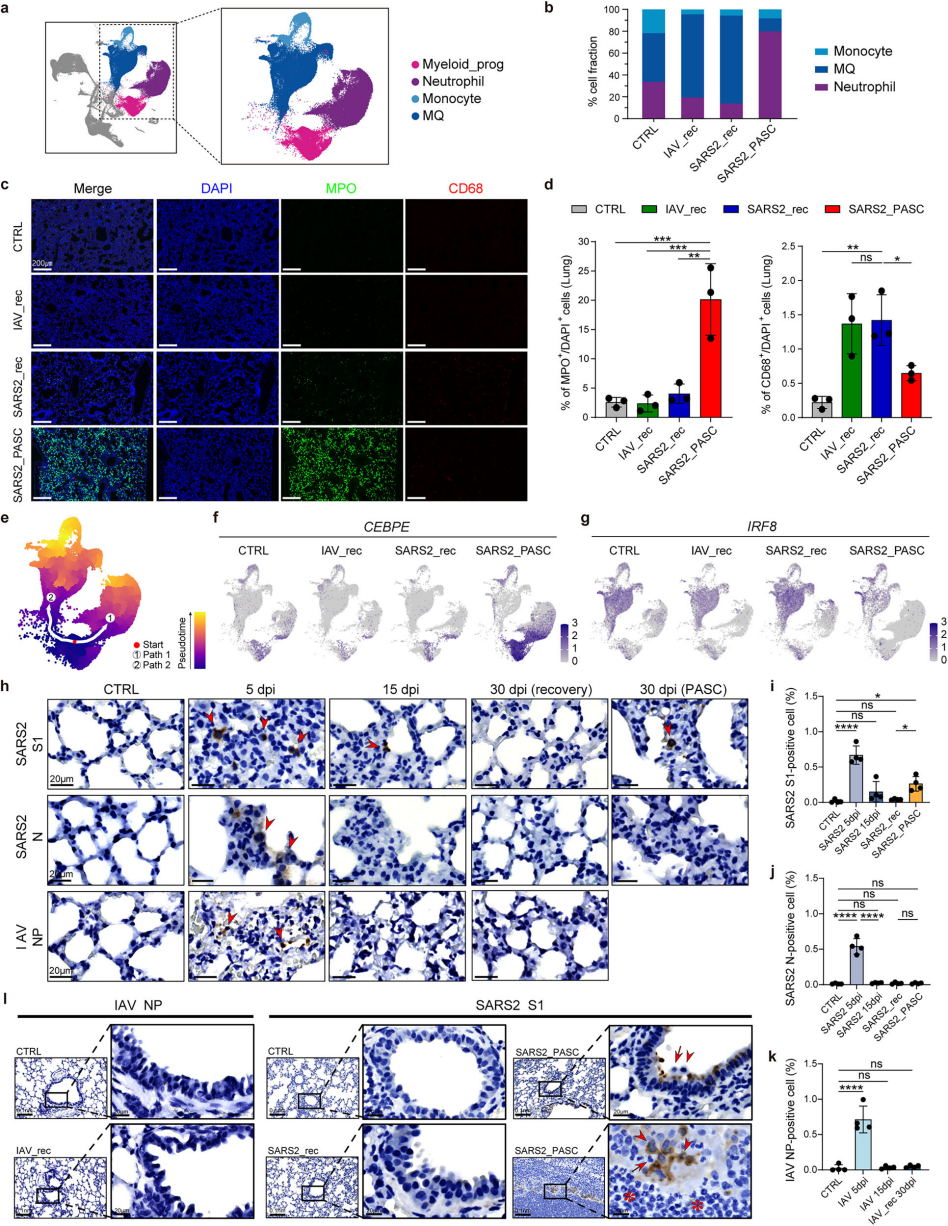
Altered myeloid differentiation and persistent neutrophil accumulation in the SARS2_PASC group, with prolonged presence of S1 antigen. **a** UMAP plot showing myeloid cell populations. **b** Proportions of MQ, monocyte and neutrophil populations across different groups. **c** Multiplex immunofluorescence image of 30 dpi lung tissues. Each group of tissues was stained with DAPI (nuclei, blue), MPO (green) and CD68 (red) (scale bars, 200 μm). **d** Quantification of the percentage of MPO^+^ and CD68^+^ cells in 30 dpi lung tissues (*n* = 3). **e** Trajectory analysis depicting the differentiation of myeloid progenitor cells into either monocytes/MQ or neutrophils with Monocle3. The UMAP is colored by pseudotime. Arrows 1 and 2 indicate distinct differentiation paths. **f**, **g** Feature plots showing *CEBPE* (**f**) and *IRF8* (**g**) gene expression in each group, respectively. **h** Representative images showing IHC results of viral antigens in lung tissue. Arrowheads indicate antigen-positive cells (scale bars, 20 μm). **i**–**k** Quantification of the percentage of SARS-CoV-2 (SARS2) S1-positive (**i**), N-positive (**j**) and IAV NP-positive (**k**) cells in lung tissue at different dpi. **l** Magnified images (scale bars, 20 μm) comparing the presence of antigens and immune cells at 30 dpi. Arrowheads indicate monocytes/MQs, arrows indicate neutrophils and asterisks highlight areas of inflammation. Data are presented as means ± s.d. (**d** and **i**–**k**). Statistical significance is indicated as follows: *P < 0.05, **P < 0.01, ***P < 0.001, ****P < 0.0001, ns (*P* > 0.05). The *P* values were estimated using one-way ANOVA. Myeloid_prog, myeloid progenitor cells; CTRL, control group; IAV_rec, IAV_recovery group; SARS2_rec, SARS-CoV-2_recovery group; SARS2_PASC, SARS-CoV-2_non-recovery group; S1, spike protein S1 subunit; N, nucleocapsid; NP, nucleoprotein.

scRNA-seq analysis revealed a marked increase in neutrophil levels and a corresponding decrease in MQ levels in the BALF and lung tissues of the SARS2_PASC group (Fig. 3a, b). To validate the distinct cell populations observed in the scRNA-seq data, we performed multiplex immunofluorescence on hamster lung tissues (Fig. 3c). Analysis of the percentage of MPO-positive cells (neutrophil marker) and CD68-positive cells (monocyte/MQ marker) revealed that the SARS2_PASC group exhibited more than a fivefold increase in neutrophils compared with the recovery groups, along with a significant decrease in CD68-positive cells (Fig. 3d). These results corroborate the scRNA-seq findings, establishing elevated neutrophil levels as a hallmark feature of the SARS2_PASC group.

To investigate the mechanisms underlying the increased neutrophil levels and decreased MQ levels in the SARS2_PASC group, we conducted a trajectory analysis of myeloid cell populations in the UMAP plots to investigate their compositional differences (Fig. 3e). To this end, myeloid progenitors were classified into granulocyte–monocyte progenitor (GMP), neutrophil progenitor (Neu_prog) and monocyte progenitor (Mono_prog) subtypes using eight marker genes (Supplementary Fig. 3a,b). Our trajectory analysis identified two distinct differentiation pathways: GMP to neutrophils via Neu_prog (path1) and GMP to monocytes/MQs via Mono_prog (path2) (Fig. 3e and Supplementary Fig. 3c). Accordingly, neutrophil-specific transcription factors (TFs) were highly expressed in Neu_prog populations, while monocyte/MQ-specific TFs were more prominent in Mono_prog populations (Supplementary Fig. 3d). Notably, in the SARS2_PASC group, the GMP, Neu_prog and neutrophil populations exhibited increased expression of neutrophil-specific TFs, particularly *CEBPE*, which is critical for neutrophil maturation, along with other key TFs such as *KLF5* and *GFI1*^32–35^ (Fig. 3f and Supplementary Fig. 3e, f). Conversely, TFs associated with monocyte/MQ differentiation, such as *IRF8*^36^, were substantially downregulated in the SARS2_PASC group compared with recovery groups, which had higher monocyte and MQ ratios (Fig. 3g). These findings suggest that the skewed neutrophil-to-monocyte/MQ ratios observed in the SARS2_PASC group are driven by disrupted myeloid cell differentiation pathways.

### Persistent neutrophil accumulation in the SARS2_PASC group: the role of residual S1 antigen

Although both SARS-CoV-2 and IAV infection groups effectively suppressed viral replication by 13 dpi, the persistent neutrophil accumulation and reduced MQ population observed exclusively in the SARS2_PASC group remain unexplained. We hypothesized that, despite the absence of detectable viral RNA, other factors might drive sustained tissue inflammation in this group. Recent studies have suggested that the SARS-CoV-2 spike protein, particularly the S1 subunit, can persist in tissues and contribute to prolonged sequelae, even in the absence of viral RNA^37,38^. To test this hypothesis, we investigated the presence of viral antigens, specifically the S1 subunit, which is known to modulate key TFs involved in immune response regulation^39,40^. IHC revealed that S1 antigens remained detectable in the SARS2_PASC group up to 30 dpi but were absent in the SARS2_rec group (Fig. 3h, i and Supplementary Fig. 3g). By contrast, SARS-CoV-2 nucleocapsid (N) and IAV nucleoprotein (NP) were detectable only up to 5 dpi and completely cleared by 15 dpi (Fig. 3h, j, k and Supplementary Fig. 3g). In the SARS2_PASC group, the S1 antigens were distributed within interstitial pneumonia lesions, accompanied by inflammatory cell infiltration, indicative of ongoing inflammation (Fig. 3h, l and Supplementary Fig. 3g). Notably, monocytes/MQs were observed to internalize S1 antigens, with neutrophils infiltrating the surrounding areas (Fig. 3l, arrowheads and asterisks). These findings highlight a distinct pattern of chronic inflammation in SARS-CoV-2-infected tissues, driven by the prolonged presence of S1 antigens, a phenomenon not observed in IAV-infected tissues, where NP was cleared much earlier. This finding underscores a critical pathophysiological difference between SARS-CoV-2 and IAV infections. While both viruses achieve similar replication suppression timelines, the residual S1 antigens in SARS-CoV-2-infected tissues appear to sustain inflammatory responses, consistent with previous findings^38,41^. Moreover, the prolonged presence of S1 antigens may influence TF expression, skewing immune responses and contributing to the non-recovery phenotype observed in the SARS2_PASC group. These results provide key insights into the mechanisms underlying PASC and suggest that persistent viral antigens, rather than active replication, drive chronic inflammation in this condition.

### Distinct myeloid subpopulations and their contribution to inflammation and fibrosis in PASC

Consistent with histopathological findings of persistent fibrosis in the SARS2_PASC group (Fig. 1n,o), we observed dense infiltration of MPO- and CD68-positive cells in fibrotic lung regions (Supplementary Fig. 4a). Transcriptional profiling revealed that neutrophils, monocytes and MQs exhibited significantly elevated module scores for inflammation and fibrosis-related gene sets (Supplementary Fig. 4b). Key mediators such as *S100A8*, *S100A9*, *IL1B*, *MMP9*, *CSF1*, *TGFA* and *TGFB1* were strongly upregulated in these cell types (Supplementary Fig. 4c). CellChat analysis further identified myeloid populations as major contributors to TGF-β signaling^25,42^, with enriched interactions both within the myeloid compartment and between myeloid and alveolar epithelial cells^43–45^ (Supplementary Fig. 4d). These findings suggest that enhanced cross-talk among myeloid cells and their stromal environment promotes fibrotic remodeling in SARS2_PASC lungs.

**Fig. 4:**
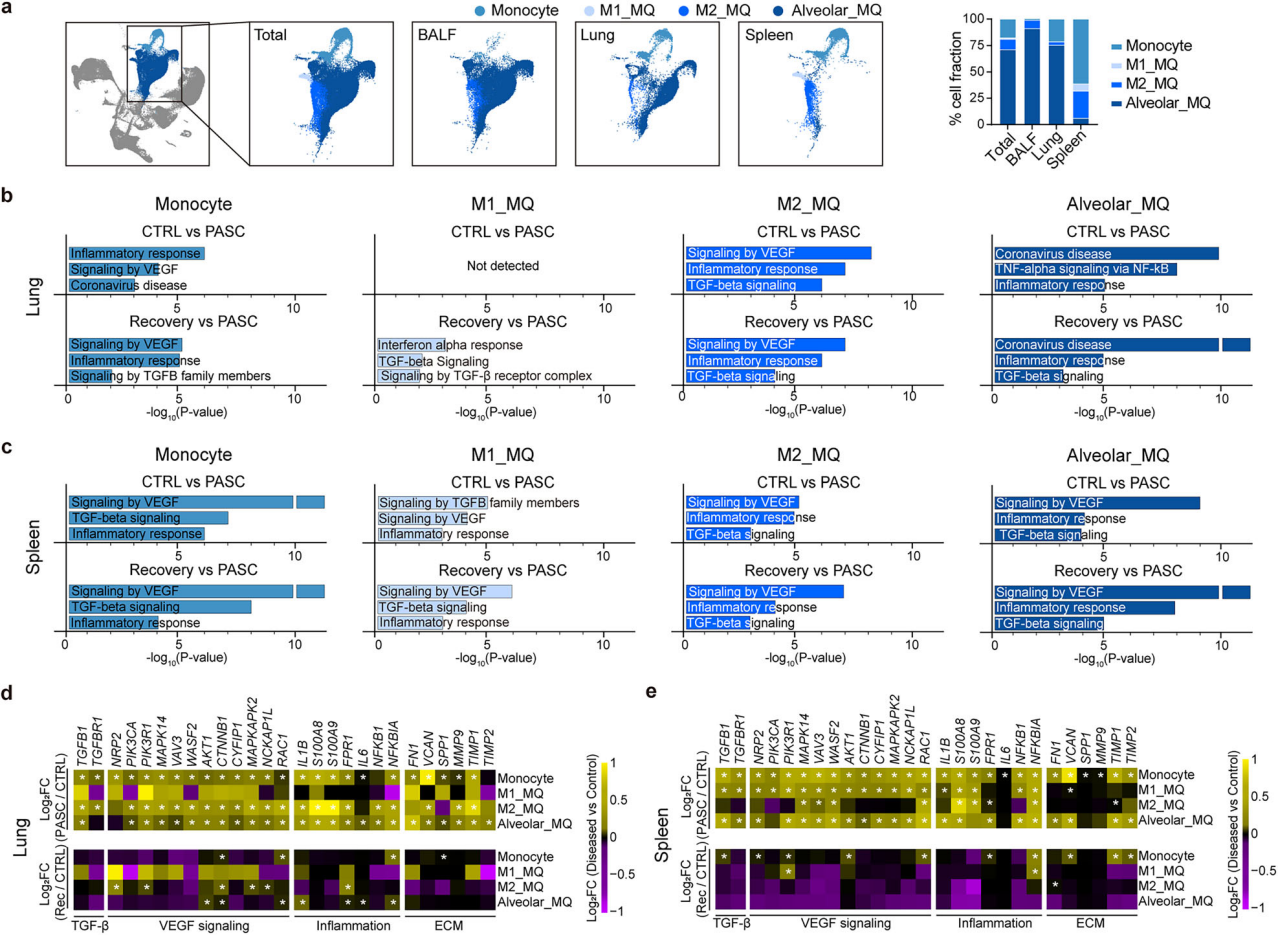
Characterization of myeloid cell populations and comparative gene expression patterns. **a** UMAP plot and distribution ratios of monocyte and MQ subpopulations across tissues. **b**, **c** Bar plots displaying −log_10_(*P* value) from EnrichR analysis with MSigDB Hallmark, GOBP, KEGG and Reactome databases. The analysis focuses on specifically elevated genes in the SARS2_PASC group compared with control (CTRL) or the IAV_ and SARS-CoV-2_ recovery (recovery) group in monocytes and MQ subpopulations. Data are presented for lung (**b**) and spleen (**c**) tissues. **d**, **e** Heatmap displaying the log_2_FC values of fibrosis-related pathway and inflammation genes in monocyte and MQ subpopulations of the lung (**d**) or spleen (**e**) tissue. log_2_FC values represent the average expression levels of each gene in the diseased groups compared to the control and are depicted by color intensity. Significance was determined using the Wilcoxon rank-sum test, and all *P* < 0.05 are represented with an asterisk owing to space limitations. M1_MQ, M1 type macrophages; M2_MQ, M2 type macrophages; Alveolar_MQ, alveolar macrophages.

Given the important roles of myeloid populations in lung fibrosis, to further investigate the characteristics of MQ differentiation from myeloid cells during SARS-CoV-2 infection, we classified MQs into three distinct subpopulations: M1 MQs (M1_MQ), M2 MQs (M2_MQ) and alveolar MQs (Alveolar_MQ) (Fig. 4a and Supplementary Fig. 4e). In respiratory tissues such as BALF and lung, alveolar MQs dominated the MQ population, comprising 75.30–91.30% of total MQs. Conversely, monocytes, M1_MQ and M2_MQ were more prevalent in the spleen, highlighting tissue-specific distribution patterns of MQ subsets (Fig. 4a). Comparative functional analyses revealed substantially heightened inflammatory responses in monocytes and MQs of the SARS2_PASC group compared with recovery (SARS2_rec, IAV_rec) and CTRL groups (Fig. 4b, c and Supplementary Table 5). Notably, monocytes, M2_MQ and alveolar MQs in the lungs of the SARS2_PASC group exhibited upregulation of genes associated with TGF-β, VEGF and inflammation pathways, indicating their active involvement in localized inflammation and fibrosis. By contrast, M1_MQ populations in the spleen displayed more pronounced engagement in these pathways, reflecting tissue-specific functional divergence (Fig. 4c).

Importantly, monocytes, M2_MQ and alveolar MQs in the lungs of the SARS2_PASC group persistently overexpressed key extracellular matrix (ECM) genes, including *FN1*, *VCAN*, *TIMP1* and *TIMP2*, as well as *TGFB1*, a critical profibrotic cytokine that stimulates ECM production^42,46^ (Fig. 4d and Supplementary Fig. 4f). These genes are closely linked to fibrosis and tissue remodeling, suggesting that these MQ subpopulations are central to sustained ECM deposition and fibrotic progression. Furthermore, fibrosis-associated pathways were highly upregulated in monocytes and M2_MQ populations of the SARS2_PASC group compared with the CTRL group, emphasizing their role in perpetuating lung fibrosis during PASC. In addition, monocytes and M2_MQs in the lungs and spleen of the SARS2_PASC group showed elevated expression of inflammation-related genes, such *S100A8*, *S100A9* and *FPR1* (Fig. 4d, e and Supplementary Fig. 4f, g). This suggests that these subpopulations actively contribute to the inflammatory milieu observed in PASC, further differentiating the SARS2_PASC group from the recovery and control groups.

### Hyperactivated neutrophil subpopulations in the SARS2_PASC group

**Fig. 5:**
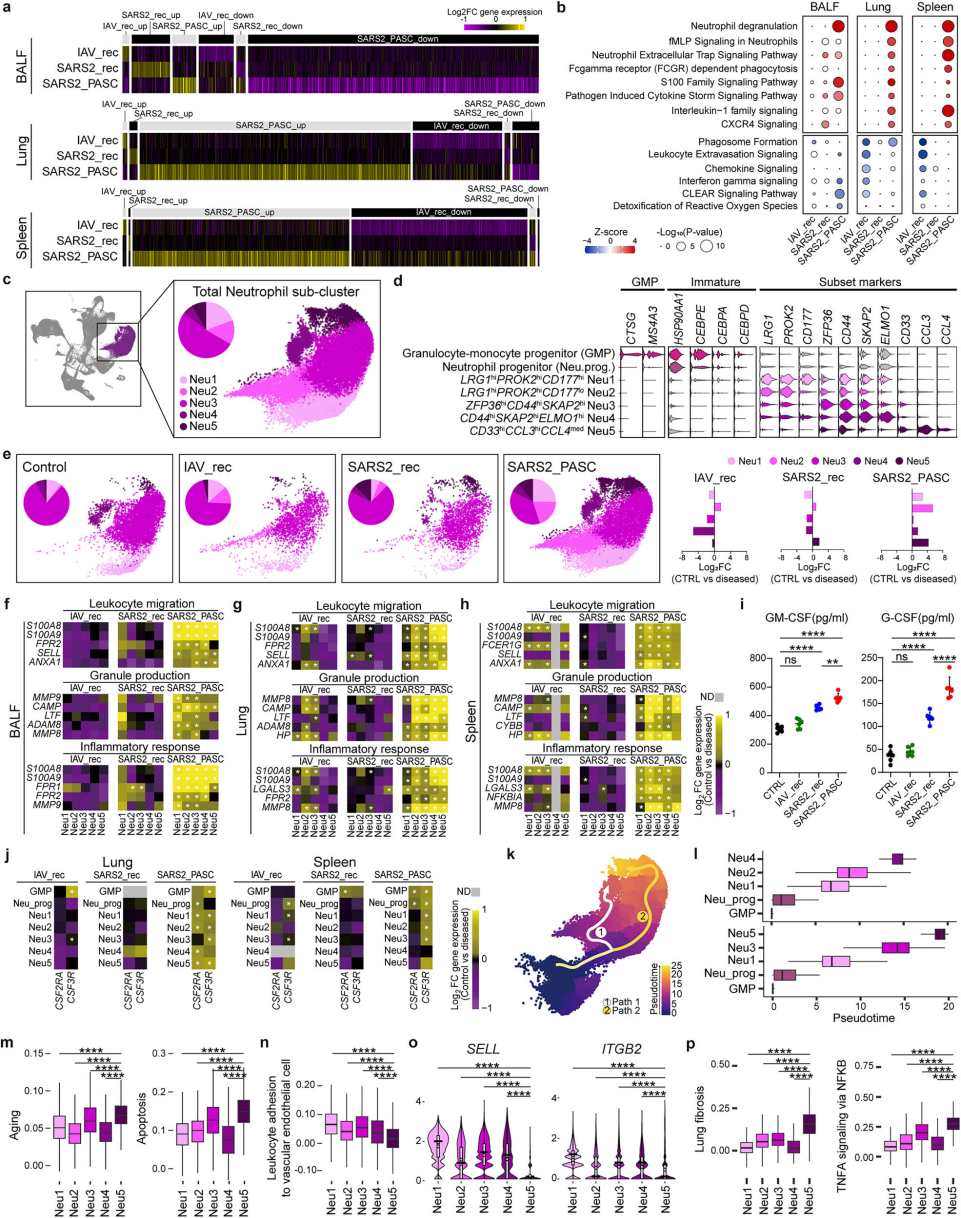
Gene expression and functional analysis of neutrophil subclusters. **a** Heat maps displaying notable upregulated or downregulated genes in neutrophils compared with the control group. log_2_FC values of average expression per gene are represented with colors ranging from high (yellow) to low (purple). **b** Dot plot of the IPA canonical pathways analysis showing the −log_10_(*P* value) (dot size) and the *Z* score (color), in diseased groups compared with control. **c** UMAP and pie chart depicting the total neutrophil subcluster population. **d** Multiple violin plot illustrating the expression levels of specific marker genes in the different neutrophil subclusters. **e** UMAPs and pie charts (left) displaying the population distribution of each neutrophil subcluster for each group. Bar plots (right) showing relative population changes are noted in all diseased groups compared with the control group. **f**–**h** Heat maps of neutrophil function-related genes in BALF (**f**), lung (**g**) and spleen (**h**). The log_2_FC expression values of each gene in the diseased groups compared with the control group is represented by color. **i** GM-CSF and G-CSF levels measured via ELISA in hamster serum at 30 dpi across all groups (*n* = 6). Data are presented as means ± s.d. **j** Heat maps of relative gene expression levels of *CSF2RA* and *CSF3R* in lung or spleen tissues. log_2_FC values of gene expression are calculated for GMP, Neu_prog and neutrophil subclusters, comparing the diseased groups with control group. **k** Trajectory analysis of myeloid progenitors and neutrophil subclusters showing two differentiation paths from GMP to neutrophil subclusters. **l** Box plots displaying the pseudotime distribution of each cell in the subclusters along the two differentiation paths. **m**, **n** Box plots displaying module scores for aging and apoptosis-related gene expression (**m**), and leukocyte adhesion to vascular endothelial cell (**n**) in neutrophil subclusters; aging (de Magalhaes et al. [24]), apoptosis (M5902) and leukocyte adhesion to vascular endothelial cells (M14170, GO:0061756). **o** Violin plots showing the expression levels of *SELL* and *ITGB2*, key genes involved in leukocyte adhesion to vascular endothelial cells. **p** Box plots showing module scores of lung fibrosis (WP3624) and TNFA signaling via NFKB (M5890) in neutrophil subclusters. Some significance was determined using the Wilcoxon rank-sum test, and all *P* < 0.05 are represented with an asterisk owing to space limitations (**f**–**h** and **j**). Other statistical significances are indicated as follows: *P < 0.05, **P < 0.01, ***P < 0.001, ****P < 0.0001, ns (*P* > 0.05). The *P* values were calculated using Wilcoxon rank-sum test (**f**–**h**, **j** and **m**–**p**) and one-way ANOVA (**i**). CTRL, control group; IAV_rec, IAV_recovery group; SARS2_rec, SARS-CoV-2_recovery group; SARS2_PASC, SARS-CoV-2_non-recovery group.

To investigate the mechanisms underlying the dramatic increase in neutrophils observed in the SARS2_PASC group, we analyzed genes that were specifically upregulated or downregulated in each group compared to CTRL (Fig. 5a). IPA revealed that neutrophils in the lung and spleen of the SARS2_PASC group exhibited high activation of pathways such as ‘formyl-methionyl-leucyl-phenylalanine (fMLP) signaling’, ‘neutrophil extracellular trap signaling’ and ‘interleukin-1 family signaling’ pathways (Fig. 5b). In the BALF, additional pathways including ‘S100 family signaling’ and ‘pathogen-induced cytokine storm signaling’ were notably upregulated. Consistent upregulation of the ‘neutrophil degranulation’ pathway across all three tissues further indicated a state of heightened inflammatory response and immune activation mediated by neutrophils in the SARS2_PASC group. By contrast, the recovery groups showed minimal changes in inflammation-related genes (Fig. 5a, b), indicating resolved inflammation. Interestingly, the IAV_rec group showed reduced activation of ‘leukocyte extravasation signaling’ and ‘chemokine signaling’ in the lungs and spleens compared with both the SARS2_rec and SARS2_PASC groups, underscoring shared yet distinct immune responses between IAV and SARS-CoV-2 infections.

To explore functional differences among neutrophils, we further categorized them into five subclusters (Neu1–Neu5) based on marker genes associated with neutrophil maturation and function (Fig. 5c, d). Cell distribution analysis revealed that neutrophil numbers decreased in both the IAV_rec and SARS2_rec groups compared with the CTRL group (Fig. 5e). Conversely, all neutrophil subclusters increased markedly in the SARS2_PASC group. Unlike their counterparts in the recovery groups, neutrophils in the SARS2_PASC group (Neu1–Neu5) displayed high expression of genes associated with ‘leukocyte migration’, ‘granule production’ and ‘inflammatory response’, including *S100A8*, *S100A9, FPR1*, *FPR2*, *MMP8* and *MMP9* (Fig. 5f–h and Supplementary Fig. 5a–c). ELISA confirmed elevated levels of GM-CSF and G-CSF, critical for neutrophil development, survival and function, in the SARS2_PASC group^47,48^ (Fig. 5i). These increases corresponded with the upregulation of their receptors, *CSF2RA* and *CSF3R*, in all Neu1–Neu5 subsets (Fig. 5j). Collectively, these findings indicate that the coordinated upregulation of receptor-cytokine genes supports sustained neutrophil activation and inflammation in the SARS2_PASC group.

Neutrophil trajectory analysis revealed two differentiation pathways: Neu4 via Neu1 and Neu2 (path1) and Neu5 via Neu1 and Neu3 (path2) (Fig. 5k, l and Supplementary Fig. 5d). Neu4, expanding across all tissues, exhibited mature neutrophil characteristics^49^ (Fig. 5d and Supplementary Fig. 5e). By contrast, Neu5, predominantly in BALF, showed higher module scores associated with aging and cell death than Neu1-4 subsets (Fig. 5m and Supplementary Fig. 5f, g). Notably, Neu5 also had lower module scores for ‘leukocyte adhesion to vascular endothelial cells’, with reduced expression of key genes^50^ (*SELL* and *ITGB2*) (Fig. 5n, o). To further define the functional contribution of neutrophil subsets to chronic lung pathology in PASC, we compared inflammatory and fibrotic gene signatures across Neu1–Neu5 populations. Among these, Neu5 exhibited the highest enrichment for gene modules associated with lung fibrosis, TNF-α/NF-κB signaling, and inflammatory responses (Fig. 5p and Supplementary Fig. 5h). Expression of fibrosis-associated genes, including *TGFB1, HGF, IL1B, CCL3, CSF1* and *CEBPB*, was strongly elevated in Neu5 relative to other subsets (Supplementary Fig. 5i). Notably, Neu5 also showed features of cellular senescence and reduced leukocyte adhesion, suggesting impaired clearance and selective retention in BALF. These findings support Neu5 as a functionally distinct, aged neutrophil population that plays a predominant role in driving tissue damage and chronic inflammation in SARS2_PASC. These findings highlight the critical role of neutrophil dynamics in PASC pathogenesis and the therapeutic potential of targeting neutrophil-driven inflammation.

### Targeted neutrophil inhibitors effectively mitigate PASC progression in SARS-CoV-2-Infected hamsters

Neutrophil-mediated inflammation has been implicated as a central driver of PASC. Recent studies have identified key proinflammatory mediators, such as S100A8, S100A9 and their heterodimer S100A8/A9 (calprotectin)^51^, as well as FPR2, a chemotaxis regulator crucial for neutrophil migration and infiltration at infection sites^52,53^. In addition, neutrophil elastase plays a pivotal role in acute lung injury by promoting neutrophil-mediated tissue damage^54^. Based on these findings, we hypothesized that targeting these pathways could attenuate PASC progression.

**Fig. 6:**
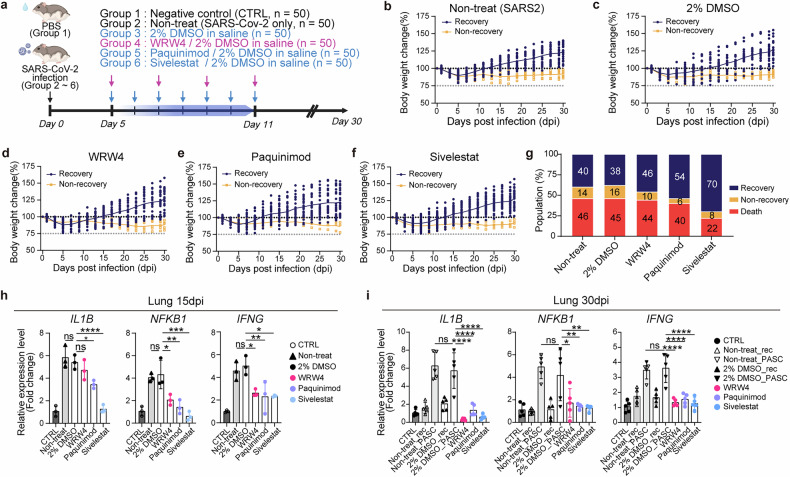
Sivelestat, a neutrophil elastase inhibitor, reduces fibrosis and inflammation in PASC. **a** A schematic overview of the animal experiment. The diagram reflects each group of the inhibitor treatment schedule. Created with BioRender.com. **b**–**f** Body weight change data of ‘Non-treat’ (SARS2, SARS-CoV-2 infection only) (**b**), 2% DMSO in a saline treatment (2% DMSO) (**c**), WRW4/2% DMSO in a saline treatment (WRW4) (**d**), Paquinimod/2% DMSO in a saline treatment (Paquinimod) (**e**) and Sivelestat/2% DMSO in a saline treatment (**f**) (*n* = 50). **g** Proportion of recovery (blue), non-recovery (yellow) and death (red) of each group. **h**, **i** Relative expression levels (indicated as delta–delta cycle threshold (Ct) values) of *IL1B*, *NFKB1* and *IFNG* at 15 (**h**) and 30 (**i**) dpi in the lung. The gene expressions were normalized by glyceraldehyde 3-phosphate dehydrogenase (*GAPDH*) as a housekeeping gene. Statistical significances are indicated as follows: *P < 0.05, **P < 0.01, ***P < 0.001, ****P < 0.0001, ns (*P* > 0.05), one-way ANOVA. CTRL, control group; Non-treat_rec, recovery group without treatment; Non-treat_PASC, non-recovery group without treatment; 2% DMSO_rec, recovery group treated with 2% DMSO in saline; 2% DMSO_PASC, non-recovery group treated with 2% DMSO in saline.

To test this hypothesis, we selected three inhibitors: Paquinimod (S100A inhibitor), WRW4 (FPR2 antagonist) and Sivelestat (NE inhibitor), each targeting key inflammatory pathways associated with PASC. SARS-CoV-2-infected *P. roborovskii* hamsters (*n* = 50 per group) were treated with these drugs from 5 to 11 dpi, during the peak pathogenic phase (Fig. 6a). Then, the recovery patterns were monitored for up to 30 dpi. The nontreated control (SARS-CoV-2 virus only) and solvent control (2% DMSO/saline) groups exhibited approximately 14.00% and 16.00% PASC incidence with over 45% mortality (Fig. 6b, c, g). By contrast, the WRW4- and Paquinimod-treated groups showed reduced PASC incidence of 10.00% and 6.00%, respectively, although mortality rates remained comparable to controls (Fig. 6d, e, g). Remarkably, the Sivelestat-treated group exhibited a 30.0% increase in recovery rate (with only an 8.0% PASC incidence) and more than a 50% reduction in mortality (22.0% mortality) (Fig. 6f, g). By contrast, delayed treatment initiated after 15 dpi (15–21 dpi) failed to enhance recovery or prevent weight loss (Supplementary Fig. 6a–c). This result suggests that irreversible pathological changes had begun to take place in a subset of animals at 15 dpi, and a clear divergence between recovering and nonrecovering individuals had already emerged. Consequently, delayed administration did not alter disease outcomes, underscoring the critical importance of timely therapeutic intervention to prevent long-term lung damage (Supplementary Fig. 6b, c).

To evaluate the anti-inflammatory effects of these inhibitors, we measured transcript levels of inflammation-related cytokines (*IL1B*, *NFKB1* and *IFNG)* in lung and spleen tissues using qRT–PCR. At 15 dpi, 4 days post-treatment, Paquinimod and Sivelestat significantly reduced cytokine expression in the lungs, with Sivelestat showing the greatest reduction, correlating with reduced early mortality (Fig. 6h). This suppression persisted, with cytokine levels remaining low at 30 dpi, 19 days after treatment discontinuation (Fig. 6i). In the spleen, all three drugs reduced *IL1B* expression at 15 dpi, while Paquinimod uniquely reduced *IFNG* expression (Supplementary Fig. 6d). By 30 dpi, inflammatory cytokine levels were significantly reduced across all treatment groups in the spleen (Supplementary Fig. 6e). Drug treatment also suppressed neutrophil-associated inflammatory genes (*FPR2*, *S100A9* and *ELANE*) across tissues, although lung-specific reductions were observed earlier, between 15 dpi and 30 dpi (Supplementary Fig. 6f–i). This difference probably reflects the localized accumulation of neutrophils in the lung, which was absent in the spleen. Among the inhibitors, Sivelestat exhibited the most robust anti-inflammatory effects in both lung and spleen, with suppression of inflammatory gene expression persisting up to 19 days post-treatment. This sustained efficacy underscores Sivelestat’s potential as a therapeutic strategy for mitigating PASC, providing a foundation for targeting neutrophil-driven inflammation to alleviate long-term complications of SARS-CoV-2 infection.

### Therapeutic targeting of neutrophil-mediated inflammation attenuates pulmonary fibrosis and chronic lung pathology

**Fig. 7:**
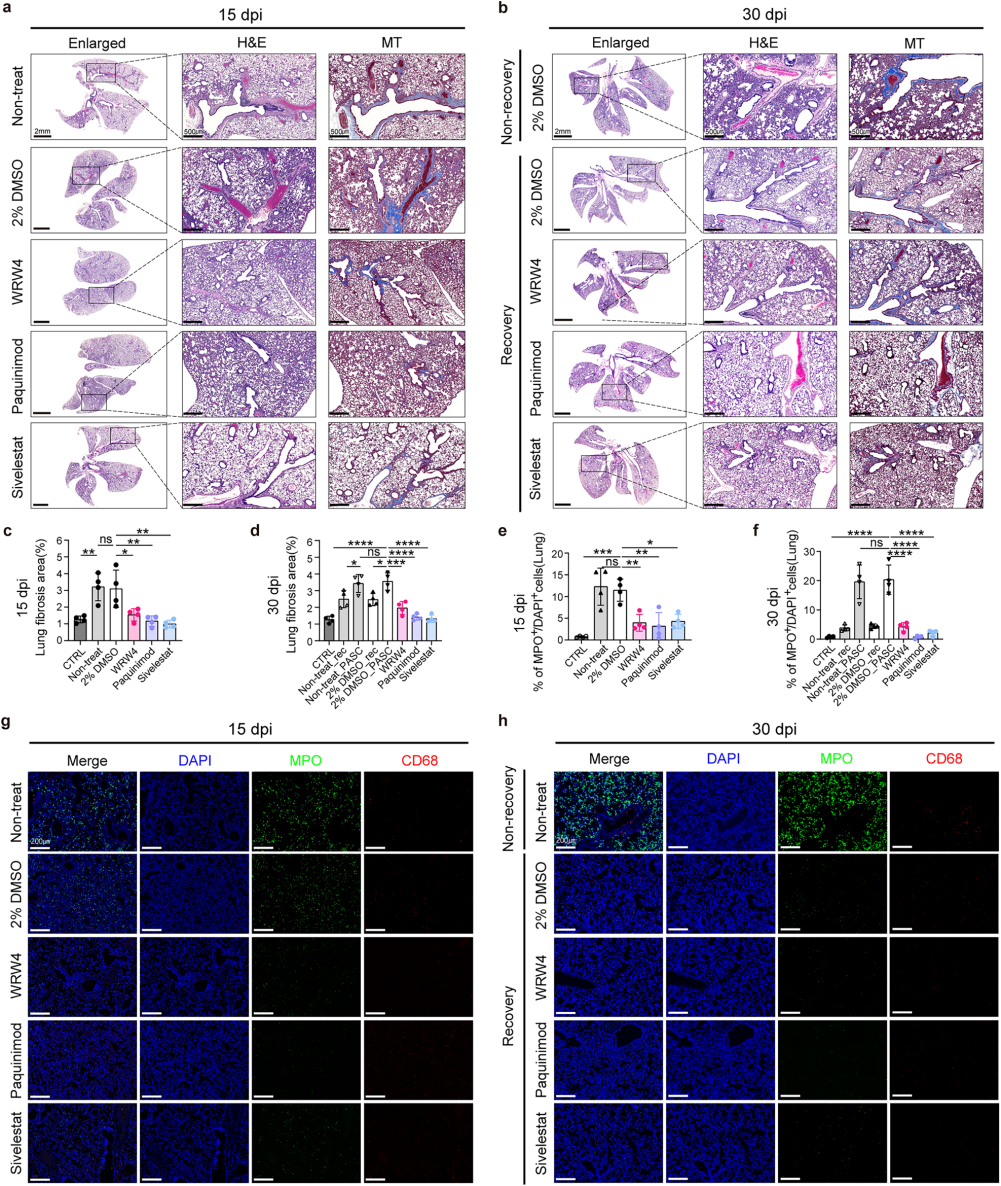
Effects of neutrophil-targeted therapies on lung pathology and inflammation. **a**, **b** H&E and MT staining image of lung tissue at 15 (**a**), 30 (**b**) dpi. Enlarged images (scale bars, 2 mm) represent total lung images of H&E, with the high-magnification section showing corresponding areas stained with H&E and MT (scale bars, 500 μm). Light-blue coloration in the MT-stained images indicates areas of fibrosis. **c**, **d** The percentage of fibrosis area relative to total lung area based on MT staining results at 15 (**c**) and 30 (**d**) dpi (*n* = 4). **e**, **f** Quantification of the percentage of MPO^+^ cells in lung tissues at 15 (**e**) and 30 (**f**) dpi (*n* = 4). **g**, **h** Multiplex immunofluorescence images of lung tissues at 15 (**g**) and 30 (**h**) dpi, stained for nuclei (DAPI, blue), MPO (green) and CD68 (red) (scale bars, 200 μm). Data for all graphs are presented as means ± s.d. Statistical significances are indicated as follows: *P < 0.05, **P < 0.01, ***P < 0.001, ****P < 0.0001, ns (*P* > 0.05), one-way ANOVA. CTRL, control group; Non-treat_rec, recovery group without treatment; Non-treat_PASC, non-recovery group without treatment; 2% DMSO_rec, recovery group treated with 2% DMSO in saline; 2% DMSO_PASC, non-recovery group treated with 2% DMSO in saline.

To evaluate the impact of drug treatment on lung pathology, lung tissues were collected at 15 and 30 dpi and subjected to histological analysis using H&E and MT staining to assess tissue morphology and fibrosis. In the nontreated and DMSO-treated groups, fibrosis was evident as early as 15 dpi and persisted through 30 dpi, indicating sustained lung damage in these groups (Fig. 7a, b). By contrast, all drug-treated groups showed significant improvements in lung pathology, with Sivelestat demonstrating the most pronounced reduction in fibrosis—exceeding 50% in cellular lesions and overall fibrotic changes at 15 dpi (Fig. 7c). By 30 dpi, fibrosis was markedly reduced across all drug-treated groups compared with untreated controls, underscoring the therapeutic efficacy of the treatments (Fig. 7d). In addition, we examined whether targeting neutrophil-associated cytokines could reduce neutrophil infiltration in the lungs. Multiplex immunofluorescence assays revealed significantly lower neutrophil levels in drug-treated groups at 15 dpi compared with the nontreated group (Fig. 7e, g). This reduction was even more pronounced by 30 dpi, with neutrophil levels decreasing by approximately 10-fold in the drug-treated groups (Fig. 7f, h). These findings confirm that targeting neutrophil-mediated inflammation with inhibitors such as Sivelestat effectively reduces neutrophil infiltration in the lungs.

**Fig. 8:**
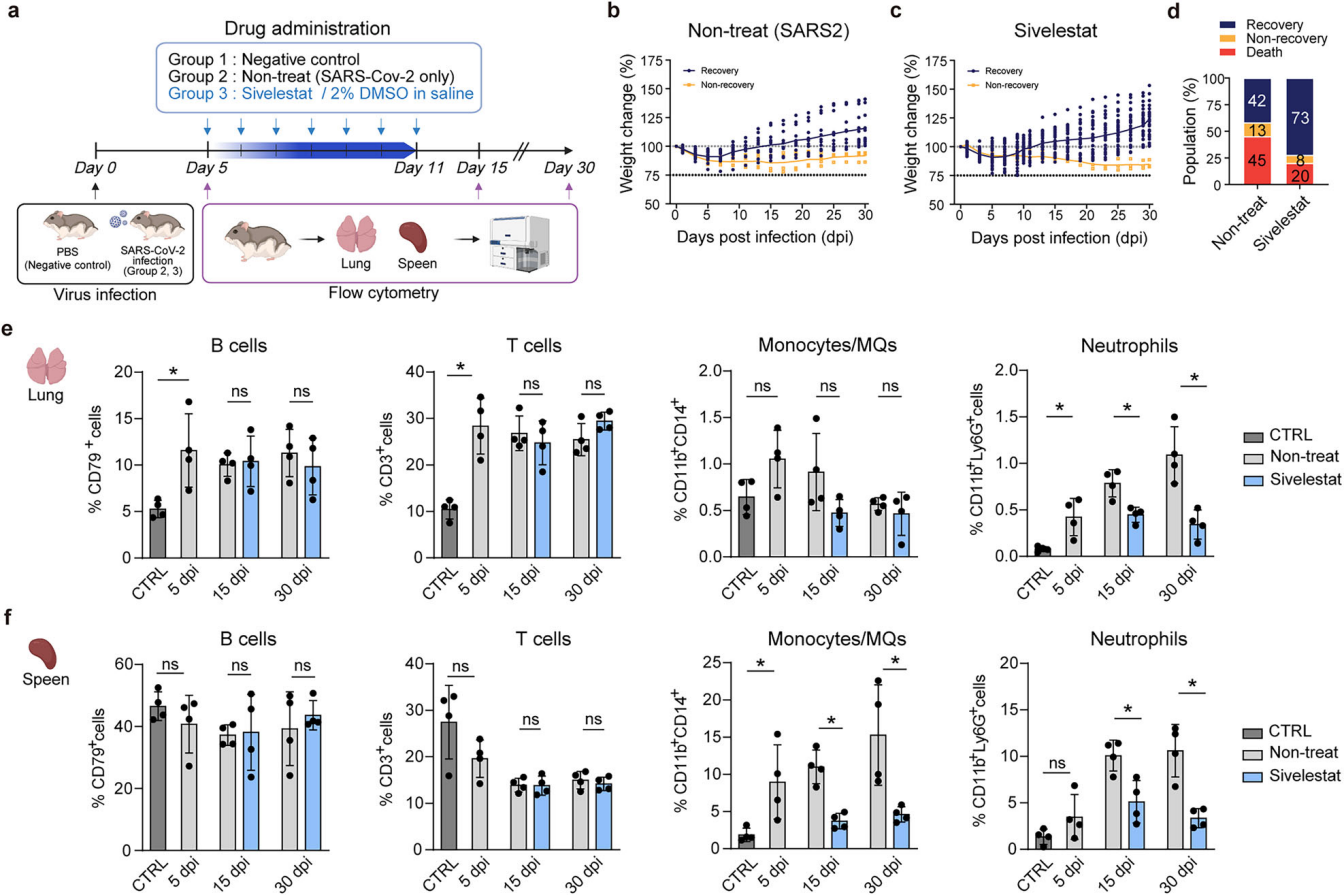
Sivelestat treatment selectively modulates innate immune cell populations in lung and spleen. **a** A schematic overview of drug administration and flow cytometry analysis. Created with BioRender.com. **b**, **c** Body weight change data of ‘Non-treat’ (SARS2, SARS-CoV-2 infection only, *n* = 40) (**b**) and Sivelestat/2% DMSO in a saline treatment (*n* = 50) (**c**). **d** Proportion of recovery (blue), non-recovery (yellow) and death (red) of each group. **e**, **f** Percentage of B cells (CD79⁺), T cells (CD3⁺), monocytes/MQs (CD11b⁺CD14⁺) and neutrophils (CD11b⁺Ly6G⁺) in the lung (**e**) and spleen (**f**). Data for all graphs are presented as means ± s.d. Statistical significances are indicated as follows: *P < 0.05, **P < 0.01, ***P < 0.001, ****P < 0.0001, ns (*P* > 0.05), Mann–Whitney test. CTRL, control group.

Interestingly, all treatment groups, Sivelestat, Paquinimod and WRW4, not only reduced the overall incidence of PASC but also decreased the detection rate of the SARS-CoV-2 S1 subunit antigen in lung tissues (Supplementary Fig. 7a, b). To further characterize the immunomodulatory effects of treatment, an additional infection study was conducted using Sivelestat, the most effective compound in reducing PASC incidence in our model, and flow cytometric analysis was performed in the lung and spleen at 5, 15 and 30 dpi (Fig. 8a and Supplementary Fig. 7c). The observed changes in body weight, recovery rates and mortality were consistent with those in the initial drug treatment experiment (Fig. 8b–d). Flow cytometry analysis revealed a marked reduction in neutrophil populations in both the lung and spleen following Sivelestat treatment, along with a significant decrease in splenic monocyte/MQ populations (Fig. 8e, f), consistent with previous reports^55^. By contrast, T and B cell frequencies remained unchanged in both organs, supporting the notion that Sivelestat exerts a selective anti-inflammatory effect primarily on the innate immune compartment.

Overall, these results highlight that therapeutic strategies targeting neutrophil activity not only reduce inflammation-associated gene expression but also markedly attenuate chronic lung pathology, including fibrosis, induced by SARS-CoV-2 infection. This approach could prove essential for managing lung damage and mitigating long-term complications associated with viral infections.

## Discussion

In this study, we developed a *P. roborovskii* hamster model that more accurately replicates human PASC symptoms compared with golden hamsters, which do not exhibit the mortality observed in severe COVID-19^56^. This model enabled us to investigate the mechanisms underlying PASC and identify potential therapeutic targets through long-term monitoring and multitissue scRNA-seq, using IAV as a comparator respiratory pathogen. Our findings revealed that, while both SARS-CoV-2 and IAV infection groups showed similar mortality rates (46.25–47.50%) and cleared viral RNA by 13 dpi, only the SARS-CoV-2-infected group had 13.75–14.00% of survivors failing to regain preinfection weight by 30 dpi, defining a SARS-CoV-2-specific phenomenon. The SARS-CoV-2 non-recovery group exhibited upregulation of hallmark PASC genes, such as *S100A8*, *S100A9*, *MMP8*, *IL1B* and *TNF* consistent with findings from human studies^28–31,56^. By contrast, this pattern was absent in the SARS-CoV-2 recovery and IAV recovery groups, indicating that the SARS-CoV-2 non-recovery group serves as a robust model for PASC-related transcriptional changes.

Systemically elevated neutrophil levels have previously been proposed as a hallmark of PASC^30,31^, but the underlying mechanisms remain unclear. Neutrophils are well recognized as first responders to acute inflammation^57^; however, recent studies highlight their emerging role in chronic inflammation, where they release proteases, form neutrophil extracellular traps and perpetuate immune activation^58,59^. Notably, our findings revealed distinct patterns of differentiation among neutrophils, monocytes, and MQs in the SARS2_PASC group. Neutrophils showed increased differentiation from GMPs, marked by overexpression of neutrophil-associated TFs (*CEBPE*,*KLF5*, and *GFI1*), while monocyte/MQ-associated TFs such as *IRF8* were downregulated. This skewed myeloid differentiation probably contributes to prolonged neutrophil infiltration and persistent inflammation^23^.

A notable discovery was the persistent presence of the SARS-CoV-2 spike protein (S1 subunit) in the lungs of the SARS2_PASC group up to 30 dpi, despite viral RNA clearance. The S1 subunit influenced transcriptional pathways related to viral infection and sustained inflammation^39,40^. Interestingly, while the SARS-CoV-2 N protein was cleared by 15 dpi, the spike S1 subunit persisted in the lung tissues of the SARS2_PASC group up to 30 dpi. This differential persistence may reflect differences in glycosylation, stability, and cellular localization. The S1 subunit is highly glycosylated, a property that enhances structural integrity and protects against proteolysis, potentially hindering its clearance by immune cells^60,61^. Furthermore, its extracellular orientation and potential interactions with immune or matrix components may allow it to persist and perpetuate inflammation, even in the absence of viral RNA^41^. This prolonged presence probably disrupted myeloid differentiation and perpetuated inflammation, contributing to the non-recovery phenotype. Monocytes/MQs internalized S1 antigens, while neutrophils infiltrated surrounding areas, fostering a state of chronic inflammation. MQ subpopulations, including monocytes, M2_MQs and alveolar MQs, exhibited persistent activation, creating proinflammatory and profibrotic environments. These activated MQs demonstrated upregulated expression of key ECM genes (for example, *FN1*, *VCAN*, *TIMP1* and *TIMP2*) and the profibrotic cytokine *TGFB1*, leading to fibrosis and tissue remodeling^42,46^. In addition, elevated expression of inflammatory genes (for example, *IL1B*, *S100A8* and *S100A9*) in MQs contributed to a potent chemoattractant landscape, amplifying neutrophil recruitment and exacerbating chronic inflammation. These findings underscore the interplay between MQs and neutrophils in driving the persistent inflammation and fibrosis characteristic of PASC-associated lung pathology, highlighting their critical roles in the disease’s progression.

Comprehensive transcriptomic analyses demonstrated a pronounced skew in myeloid development favoring hyperactivated neutrophils in the SARS2_PASC group. Even at 30 dpi, neutrophils and their progenitors showed elevated expression of key TFs (*CEBPE*), receptor genes (*CSF2RA* and *CSF3R*), alongside their cytokine counterparts (*GM-CSF* and *G-CSF*), promoting neutrophil development and activation^47,48^. These neutrophils also expressed high levels of inflammatory and tissue remodeling genes, including *S100A8*, *S100A9*, *FPR1*, *FPR2*, *MMP8* and *MMP9* which are linked to neutrophil degranulation, chronic inflammation and fibrosis. The persistent upregulation of *S100A8* and *S100A9* suggests a sustained inflammatory state, consistent with patients with PASC^56,62^. Moreover, the release of *MMPs* and neutrophil elastase contributes to ECM remodeling and pulmonary fibrosis, highlighting the critical role of hyperactivated neutrophils in PASC pathogenesis^31,63,64^. These findings align with human PASC studies linking neutrophil-driven inflammation to lung fibrosis and chronic sequelae.

To test whether targeting key mediators of neutrophil activation could mitigate PASC progression, we used inhibitors targeting FPR2 (WRW4), S100A8/A9 (Paquinimod) and neutrophil elastase (Sivelestat)^62,65,66^. While WRW4 and Paquinimod reduced PASC incidence, their impact on mortality was modest. Sivelestat, however, substantially improved outcomes, increasing recovery rates by 75%, reducing mortality by over 50% and decreasing cytokine levels, fibrosis and neutrophil infiltration in lung tissues. Neutrophil elastase plays a critical role in promoting lung fibrosis, facilitating neutrophil extracellular trap formation and enhancing neutrophil infiltration under inflammatory conditions^54,66,67^. By targeting these mechanisms, Sivelestat effectively suppressed SARS-CoV-2-induced inflammation and mitigated long-term lung damage. Although concerns have been raised regarding the potential for neutrophil elastase inhibitors to impair pathogen clearance^68^, recent clinical and preclinical data suggest that Sivelestat offers a favorable safety profile. Clinical trials in patients with acute lung injury reported no significant increase in infection rates among Sivelestat-treated groups^69^. Moreover, preclinical animal studies have shown that Sivelestat selectively inhibits extracellular NE while preserving intracellular NE activity required for microbial killing, thereby minimizing unintended immunosuppression^70^. These findings support the therapeutic potential of Sivelestat in neutrophil-driven inflammatory diseases such as PASC, although additional studies are needed to evaluate its safety and efficacy across diverse infectious and immunological contexts. These findings underscore the therapeutic potential of targeting neutrophil activation to manage PASC. Notably, administering drugs between 5 dpi and 11 dpi during the acute phase of infection effectively prevented irreversible damage and chronic symptoms. This timing minimized stress on the animals while optimizing therapeutic efficacy, offering critical insights into early intervention strategies for PASC.

This study highlights the complex interplay between neutrophils and monocytes/MQs in driving PASC-associated inflammation and fibrosis using a *P. roborovskii* hamster model. Persistent SARS-CoV-2 S1 antigen and sustained neutrophil activation were identified as key contributors to lung damage in the SARS2_PASC group. Targeting neutrophil-mediated inflammation with specific inhibitors not only reduced PASC incidence but also markedly lowered mortality, providing a strong basis for early intervention strategies. These findings establish the *P. roborovskii* hamster model as a robust tool for studying PASC and underscore the therapeutic potential of targeting neutrophil activation to manage long COVID complications.

## Supplementary information

Supplementary Information

Supplementary Table 3

Supplementary Table 4

Supplementary Table 5

## Data Availability

The scRNA-seq data generated in this Article have been deposited at the National Center for Biotechnology Information (NCBI) Gene Expression Omnibus (GEO) under accession number GSE288927.

## References

[1] Zhu, N. et al. A Novel Coronavirus from Patients with Pneumonia in China, 2019. *N. Engl. J. Med.* **382**, 727–733 (2020). 31978945 10.1056/NEJMoa2001017 PMC7092803

[2] Mishra, N. P. et al. Global impacts of pre- and post-COVID-19 pandemic: Focus on socio-economic consequences. *Sens. Int.* **1**, 100042 (2020). 34766044 10.1016/j.sintl.2020.100042 PMC7510561

[3] Mongin, D. et al. Effect of SARS-CoV-2 prior infection and mRNA vaccination on contagiousness and susceptibility to infection. *Nat. Commun.* **14**, 5452 (2023). 37673865 10.1038/s41467-023-41109-9 PMC10482859

[4] Manirambona, E., Okesanya, O. J., Olaleke, N. O., Oso, T. A. & Lucero-Prisno, D. E. III Evolution and implications of SARS-CoV-2 variants in the post-pandemic era. *Discov. Public Health* **21**, 16 (2024).

[5] Horberg, M. A. et al. Post-acute sequelae of SARS-CoV-2 with clinical condition definitions and comparison in a matched cohort. *Nat. Commun.* **13**, 5822 (2022). 36224218 10.1038/s41467-022-33573-6 PMC9556630

[6] Wang, C., Ramasamy, A., Verduzco-Gutierrez, M., Brode, W. M. & Melamed, E. Acute and post-acute sequelae of SARS-CoV-2 infection: a review of risk factors and social determinants. *Virol. J.* **20**, 124 (2023). 37328773 10.1186/s12985-023-02061-8 PMC10276420

[7] Thaweethai, T. et al. Development of a definition of postacute sequelae of SARS-CoV-2 infection. *Jama* **329**, 1934–1946 (2023). 37278994 10.1001/jama.2023.8823 PMC10214179

[8] Cheon, I. S. et al. Immune signatures underlying post-acute COVID-19 lung sequelae. *Sci. Immunol.* **6**, eabk1741 (2021). 34591653 10.1126/sciimmunol.abk1741 PMC8763087

[9] Yoon, H. et al. Single-cell RNA sequencing reveals characteristics of myeloid cells in post-acute sequelae of SARS-CoV-2 patients with persistent respiratory symptoms. *Front. Immunol.* **14**, 1268510 (2023). 38259488 10.3389/fimmu.2023.1268510 PMC10800799

[10] Trimpert, J. et al. The Roborovski dwarf hamster is a highly susceptible model for a rapid and fatal course of SARS-CoV-2 infection. *Cell Rep.* **33**, 108488 (2020). 33271063 10.1016/j.celrep.2020.108488 PMC7674129

[11] Song, M. S. et al. Increased virulence of neuraminidase inhibitor-resistant pandemic H1N1 virus in mice: potential emergence of drug-resistant and virulent variants. *Virulence* **4**, 489–493 (2013). 23924955 10.4161/viru.25952 PMC5359727

[12] Kim, E. H. et al. Coinfection with SARS-CoV-2 and influenza A virus increases disease severity and impairs neutralizing antibody and CD4^+^ T cell responses. *J. Virol.* **96**, e0187321 (2022). 35107382 10.1128/jvi.01873-21 PMC8941868

[13] Ilyushina, N. A. et al. Adaptation of pandemic H1N1 influenza viruses in mice. *J. Virol.* **84**, 8607–8616 (2010). 20592084 10.1128/JVI.00159-10 PMC2918990

[14] Reed, L. J. & Muench, H. A simple method of estimating fifty per cent endpoints. *Am. J. Epidemiol* **27**, 493–497 (1938).

[15] Huerta-Cepas, J. et al. eggNOG 5.0: a hierarchical, functionally and phylogenetically annotated orthology resource based on 5090 organisms and 2502 viruses. *Nucleic Acids Res.* **47**, D309–d314 (2019). 30418610 10.1093/nar/gky1085 PMC6324079

[16] Hao, Y. et al. Integrated analysis of multimodal single-cell data. *Cell* **184**, 3573–3587 (2021). 34062119 10.1016/j.cell.2021.04.048 PMC8238499

[17] Park, D. et al. Differential beta-coronavirus infection dynamics in human bronchial epithelial organoids. *J. Med. Virol.* **96**, e29600 (2024). 38591240 10.1002/jmv.29600

[18] Young, M. D. & Behjati, S. SoupX removes ambient RNA contamination from droplet-based single-cell RNA sequencing data. *Gigascience* **9**, giaa151 (2020). 33367645 10.1093/gigascience/giaa151 PMC7763177

[19] McGinnis, C. S., Murrow, L. M. & Gartner, Z. J. DoubletFinder: doublet detection in single-cell RNA sequencing data using artificial nearest neighbors. *Cell Syst.* **8**, 329–337 (2019). 30954475 10.1016/j.cels.2019.03.003 PMC6853612

[20] Hafemeister, C. & Satija, R. Normalization and variance stabilization of single-cell RNA-seq data using regularized negative binomial regression. *Genome Biol.* **20**, 296 (2019). 31870423 10.1186/s13059-019-1874-1 PMC6927181

[21] Cao, J. et al. The single-cell transcriptional landscape of mammalian organogenesis. *Nature* **566**, 496–502 (2019). 30787437 10.1038/s41586-019-0969-x PMC6434952

[22] Subramanian, A. et al. Gene set enrichment analysis: a knowledge-based approach for interpreting genome-wide expression profiles. *Proc. Natl Acad. Sci. USA* **102**, 15545–15550 (2005). 16199517 10.1073/pnas.0506580102 PMC1239896

[23] Evrard, M. et al. Developmental analysis of bone marrow neutrophils reveals populations specialized in expansion, trafficking, and effector functions. *Immunity* **48**, 364–379.e368 (2018). 29466759 10.1016/j.immuni.2018.02.002

[24] de Magalhães, J. P. et al. Human Ageing Genomic Resources: updates on key databases in ageing research. *Nucleic Acids Res.* **52**, D900–d908 (2024). 37933854 10.1093/nar/gkad927 PMC10767973

[25] Jin, S., Plikus, M. V. & Nie, Q. CellChat for systematic analysis of cell–cell communication from single-cell transcriptomics. *Nat. Protoc.* **20**, 180–219 (2025). 39289562 10.1038/s41596-024-01045-4

[26] Kuleshov, M. V. et al. Enrichr: a comprehensive gene set enrichment analysis web server 2016 update. *Nucleic Acids Res.* **44**, W90–W97 (2016). 27141961 10.1093/nar/gkw377 PMC4987924

[27] Patton, M. J. et al. Characteristics and determinants of pulmonary long COVID. *JCI Insight* **9**, e177518 (2024). 38652535 10.1172/jci.insight.177518 PMC11141907

[28] Cervia-Hasler, C. et al. Persistent complement dysregulation with signs of thromboinflammation in active Long COVID. *Science* **383**, eadg7942 (2024). 38236961 10.1126/science.adg7942

[29] Holms, R. D. Long COVID (PASC) is maintained by a self-sustaining pro-inflammatory TLR4/RAGE-loop of S100A8/A9> TLR4/RAGE signalling, inducing chronic expression of IL-1b, IL-6 and TNFa: anti-inflammatory ezrin peptides as potential therapy. *Immuno* **2**, 512–533 (2022).

[30] Woodruff, M. C. et al. Chronic inflammation, neutrophil activity, and autoreactivity splits long COVID. *Nat. Commun.* **14**, 4201 (2023). 37452024 10.1038/s41467-023-40012-7 PMC10349085

[31] George, P. M. et al. A persistent neutrophil-associated immune signature characterizes post-COVID-19 pulmonary sequelae. *Sci. Transl. Med.* **14**, eabo5795 (2022). 36383686 10.1126/scitranslmed.abo5795

[32] Kwok, I. et al. Combinatorial single-cell analyses of granulocyte-monocyte progenitor heterogeneity reveals an early uni-potent neutrophil progenitor. *Immunity* **53**, 303–318 (2020). 32579887 10.1016/j.immuni.2020.06.005

[33] Shyamsunder, P. et al. Identification of a novel enhancer of CEBPE essential for granulocytic differentiation. *Blood* **133**, 2507–2517 (2019). 30952671 10.1182/blood.2018886077

[34] Shahrin, N. H., Diakiw, S., Dent, L. A., Brown, A. L. & D’Andrea, R. J. Conditional knockout mice demonstrate function of Klf5 as a myeloid transcription factor. *Blood* **128**, 55–59 (2016). 27207790 10.1182/blood-2015-12-684514

[35] Hock, H. et al. Intrinsic requirement for zinc finger transcription factor Gfi-1 in neutrophil differentiation. *Immunity* **18**, 109–120 (2003). 12530980 10.1016/s1074-7613(02)00501-0

[36] Tamura, T., Kurotaki, D. & Koizumi, S. Regulation of myelopoiesis by the transcription factor IRF8. *Int. J. Hematol.* **101**, 342–351 (2015). 25749660 10.1007/s12185-015-1761-9

[37] Rong, Z. et al. Persistence of spike protein at the skull–meninges–brain axis may contribute to the neurological sequelae of COVID-19. *Cell Host Microbe* **32**, 2112–2130 (2024). 39615487 10.1016/j.chom.2024.11.007

[38] Swank, Z. et al. Persistent Circulating severe acute respiratory syndrome coronavirus 2 spike is associated with post-acute coronavirus disease 2019 sequelae. *Clin. Infect. Dis.* **76**, e487–e490 (2023). 36052466 10.1093/cid/ciac722 PMC10169416

[39] Han, H. et al. Transcriptional regulation of SARS-CoV-2 receptor ACE2 by SP1. *eLife* **13**, e85985 (2024). 38375778 10.7554/eLife.85985 PMC10878691

[40] Freitas, R. S., Crum, T. F. & Parvatiyar, K. SARS-CoV-2 spike antagonizes innate antiviral immunity by targeting interferon regulatory factor 3. *Front. Cell Infect. Microbiol.* **11**, 789462 (2021). 35083167 10.3389/fcimb.2021.789462 PMC8785962

[41] Patterson, B. K. et al. Persistence of SARS CoV-2 S1 protein in CD16^+^ monocytes in post-acute sequelae of COVID-19 (PASC) up to 15 months post-infection. *Front Immunol.* **12**, 746021 (2021). 35082777 10.3389/fimmu.2021.746021 PMC8784688

[42] Meng, X. M., Nikolic-Paterson, D. J. & Lan, H. Y. TGF-β: the master regulator of fibrosis. *Nat. Rev. Nephrol.* **12**, 325–338 (2016). 27108839 10.1038/nrneph.2016.48

[43] Wendisch, D. et al. SARS-CoV-2 infection triggers profibrotic macrophage responses and lung fibrosis. *Cell* **184**, 6243–6261 (2021). 34914922 10.1016/j.cell.2021.11.033 PMC8626230

[44] Pandolfi, L. et al. Neutrophil extracellular traps induce the epithelial-mesenchymal transition: implications in post-COVID-19 fibrosis. *Front. Immunol.* **12**, 663303 (2021). 34194429 10.3389/fimmu.2021.663303 PMC8236949

[45] Willis, B. C. et al. Induction of epithelial–mesenchymal transition in alveolar epithelial cells by transforming growth factor-beta1: potential role in idiopathic pulmonary fibrosis. *Am. J. Pathol.* **166**, 1321–1332 (2005). 15855634 10.1016/s0002-9440(10)62351-6 PMC1606388

[46] Kinashi, H., Ito, Y., Sun, T., Katsuno, T. & Takei, Y. Roles of the TGF-á?VEGF-C pathway in fibrosis-related lymphangiogenesis. *Int. J. Mol. Sci* **19**, 2487 (2018). 30142879 10.3390/ijms19092487 PMC6163754

[47] Mehta, H. M., Malandra, M. & Corey, S. J. G-CSF and GM-CSF in neutropenia. *J. Immunol.* **195**, 1341–1349 (2015). 26254266 10.4049/jimmunol.1500861 PMC4741374

[48] Castellani, S. et al. G-CSF and GM-CSF modify neutrophil functions at concentrations found in cystic fibrosis. *Sci. Rep.* **9**, 12937 (2019). 31506515 10.1038/s41598-019-49419-z PMC6736848

[49] Grieshaber-Bouyer, R. & Nigrovic, P. A. Neutrophil heterogeneity as therapeutic opportunity in immune-mediated disease. *Front. Immunol.* **10**, 346 (2019). 30886615 10.3389/fimmu.2019.00346 PMC6409342

[50] Abbassi, O., Kishimoto, T. K., McIntire, L. V. & Smith, C. W. Neutrophil adhesion to endothelial cells. *Blood Cells* **19**, 245–259 (1993). 7508770

[51] Hsu, K. et al. Anti-Infective protective properties of S100 calgranulins. *Antiinflamm. Antiallergy Agents Med. Chem.* **8**, 290–305 (2009). 20523765 10.2174/187152309789838975 PMC2879674

[52] Ampomah, P. B., Moraes, L. A., Lukman, H. M. & Lim, L. H. K. Formyl peptide receptor 2 is regulated by RNA mimics and viruses through an IFN-β-STAT3-dependent pathway. *FASEB J.* **32**, 1468–1478 (2018). 29127186 10.1096/fj.201700584RR

[53] Tcherniuk, S. et al. Formyl peptide receptor 2 plays a deleterious role during influenza A virus infections. *J. Infect. Dis.* **214**, 237–247 (2016). 27034344 10.1093/infdis/jiw127

[54] Woodman, R. C., Reinhardt, P. H., Kanwar, S., Johnston, F. L. & Kubes, P. Effects of human neutrophil elastase (HNE) on neutrophil function in vitro and in inflamed microvessels. *Blood* **82**, 2188–2195 (1993). 8400269

[55] Pan, T. et al. The neutrophil elastase inhibitor, sivelestat, attenuates acute lung injury in patients with cardiopulmonary bypass. *Front. Immunol.* **14**, 1082830 (2023). 36761773 10.3389/fimmu.2023.1082830 PMC9902923

[56] Frere, J. J. et al. SARS-CoV-2 infection in hamsters and humans results in lasting and unique systemic perturbations after recovery. *Sci. Transl. Med.* **14**, eabq3059 (2022). 35857629 10.1126/scitranslmed.abq3059 PMC9210449

[57] Peiseler, M. & Kubes, P. More friend than foe: the emerging role of neutrophils in tissue repair. *J. Clin. Invest.* **129**, 2629–2639 (2019). 31205028 10.1172/JCI124616 PMC6597202

[58] Herrero-Cervera, A., Soehnlein, O. & Kenne, E. Neutrophils in chronic inflammatory diseases. *Cell Mol. Immunol.* **19**, 177–191 (2022). 35039631 10.1038/s41423-021-00832-3 PMC8803838

[59] Wan, R. et al. Neutrophil extracellular traps amplify neutrophil recruitment and inflammation in neutrophilic asthma by stimulating the airway epithelial cells to activate the TLR4/ NF-KB pathway and secrete chemokines. *Aging* **12**, 16820–16836 (2020). 32756014 10.18632/aging.103479 PMC7521522

[60] Zhang, F. et al. SARS-CoV-2 spike glycosylation affects function and neutralization sensitivity. *mBio* **15**, e0167223 (2024). 38193662 10.1128/mbio.01672-23 PMC10865855

[61] Wang, D. et al. Enhanced surface accessibility of SARS-CoV-2 omicron spike protein due to an altered glycosylation profile. *ACS Infect. Dis.* **10**, 2032–2046 (2024). 38728322 10.1021/acsinfecdis.4c00015 PMC11184558

[62] Guo, Q. et al. Induction of alarmin S100A8/A9 mediates activation of aberrant neutrophils in the pathogenesis of COVID-19. *Cell Host Microbe* **29**, 222–235 (2021). 33388094 10.1016/j.chom.2020.12.016 PMC7762710

[63] Garc¡a-Prieto, E. et al. Resistance to bleomycin-induced lung fibrosis in MMP-8 deficient mice is mediated by interleukin-10. *PLoS ONE* **5**, e13242 (2010). 20949050 10.1371/journal.pone.0013242 PMC2951918

[64] Chua, F. et al. Mice lacking neutrophil elastase are resistant to bleomycin-induced pulmonary fibrosis. *Am. J. Pathol.* **170**, 65–74 (2007). 17200183 10.2353/ajpath.2007.060352 PMC1762691

[65] Lice, I. et al. Effects of formyl peptide receptor agonists Ac(9-12) and WKYMV in in vivo and in vitro acute inflammatory experimental models. *Cells* **11**, 228 (2022). 35053343 10.3390/cells11020228 PMC8773544

[66] Chua, F. & Laurent, G. J. Neutrophil elastase: mediator of extracellular matrix destruction and accumulation. *Proc. Am. Thorac. Soc.* **3**, 424–427 (2006). 16799086 10.1513/pats.200603-078AW

[67] Okeke, E. B. et al. Inhibition of neutrophil elastase prevents neutrophil extracellular trap formation and rescues mice from endotoxic shock. *Biomaterials* **238**, 119836 (2020). 32045782 10.1016/j.biomaterials.2020.119836 PMC7075277

[68] Cole, A. M. et al. Inhibition of neutrophil elastase prevents cathelicidin activation and impairs clearance of bacteria from wounds. *Blood* **97**, 297–304 (2001). 11133774 10.1182/blood.v97.1.297

[69] Aikawa, N. et al. Reevaluation of the efficacy and safety of the neutrophil elastase inhibitor, Sivelestat, for the treatment of acute lung injury associated with systemic inflammatory response syndrome; a phase IV study. *Pulm. Pharm. Ther.* **24**, 549–554 (2011). 10.1016/j.pupt.2011.03.001 21540122

[70] Hagio, T. et al. Inhibition of neutrophil elastase reduces lung injury and bacterial count in hamsters. *Pulm. Pharm. Ther.* **21**, 884–891 (2008). 10.1016/j.pupt.2008.10.002 18992355

