# Supplementary material for: Prolonged immune activation in post-acute sequelae of SARS-CoV-2: neutrophil dynamics and therapeutic insights
Source: Exp Mol Med. 2025 Sep 24;57(9):2067–82. doi: 10.1038/s12276-025-01539-5 (PMC12508235; doi:10.1038/s12276-025-01539-5)
Supplement: Supplementary file 1 — Supplementary Information [file 12276_2025_1539_MOESM1_ESM.docx]

**Supplementary Information for**

**Prolonged Immune Activation in Post-Acute Sequelae of SARS-CoV-2: Neutrophil Dynamics and Therapeutic Insights**

Mina Yu^1, 2, *^, Suhee Hwang^1,3 *^, Hobin Jang^1, *^, Dongbin Park^1^, Jaemoo Kim^1^, Juryeon Gil^1, 2^, Jeong Ho Choi ^2^, Seung-Gyu Jang^4^, Isaac Choi^1^, Yuri Jung^1^, Woohyun Kwon^1, 2^, Se-Mi Kim^1^, Young-Il Kim^1^, Hyunjoon Kim^1^, Taehwan Oh^1^, Joo Sang Lee^3^, Min-Suk Song^2^, SangJoon Lee^5^ and Young Ki Choi^1, 2, **^

^1^Center for Study of Emerging and Re-emerging Viruses, Korea Virus Research Institute, Institute for Basic Science (IBS), Daejeon, Republic of Korea
^2^College of Medicine and Medical Research Institute, Chungbuk National University, Cheongju, Republic of Korea
^3^ Department of Metabiohealth, Sungkyun Convergence Institute, Sungkyunkwan University (SKKU), Suwon, Republic of Korea
^4^ Virus Research Resource Center, Korea Virus Research Institute, Institute for Basic Science (IBS), Daejeon, Republic of Korea
^5^Department of Biological Science, Ulsan National Institute of Science and Technology (UNIST), Ulsan, Republic of Korea
^*^These authors contributed equally
^**^Corresponding author: Young Ki Choi (choiki55@ibs.re.kr, tel. 82+10-3034-7995, Fax. 82+42-878-8059)

**This PDF file includes:**

Supplementary Fig. 1 to 7
Supplementary Table. 1, 2
Data from Supplementary Tables 3-5, which are too large to fit in a PDF, are provided as separate Excel files.

**Supplementary Figures**

**
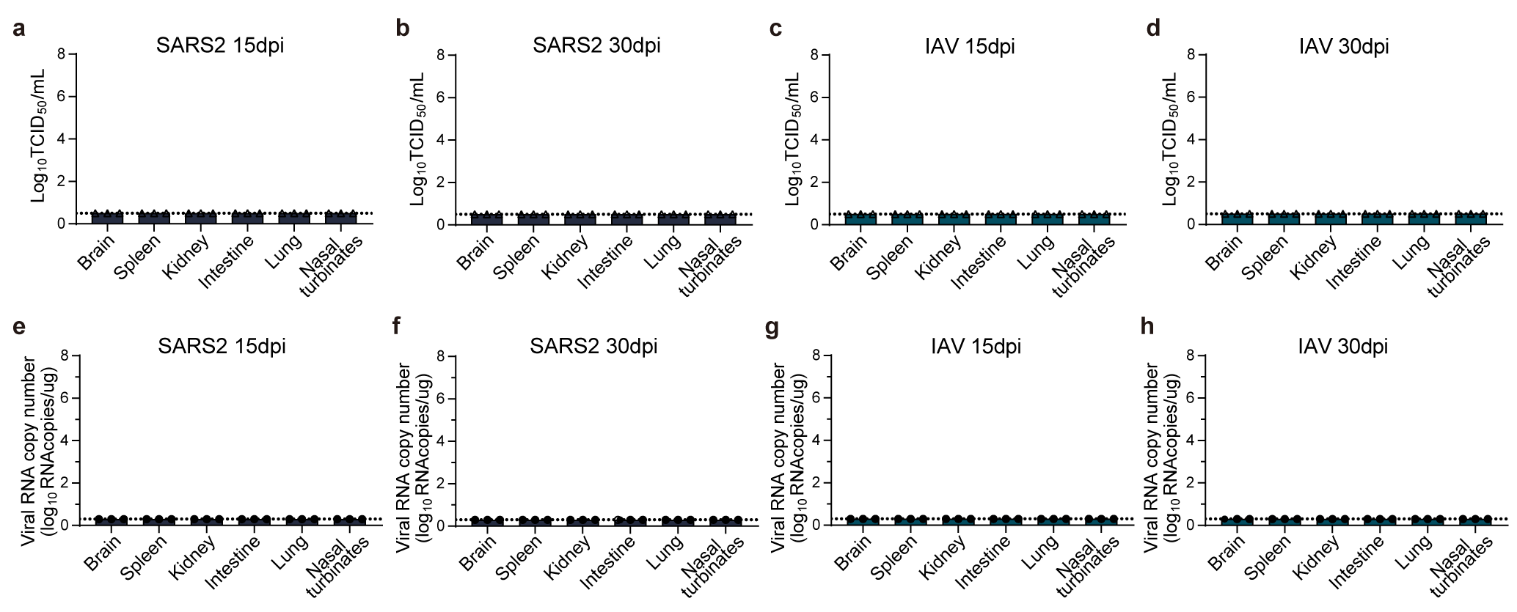
**

**Supplementary Fig. 1 Viral titer measurements in each tissue on day 15, 30 after SARS-CoV-2 or IAV infection.**
**a**–**d** TCID_50_ of various tissues in the SARS-CoV-2 infection group (SARS2) at 15(**a**), 30(**b**) dpi, and in the Influenza A virus infection group (IAV) at 15(**c**), 30(**d**) dpi (n = 3). **e**–**h** Viral RNA copy number of various tissues in SARS2 at 15(**e**), 30(**f**) dpi (n = 3), and in the IAV infection group at 15(**g**), 30(**h**) dpi (n = 3).


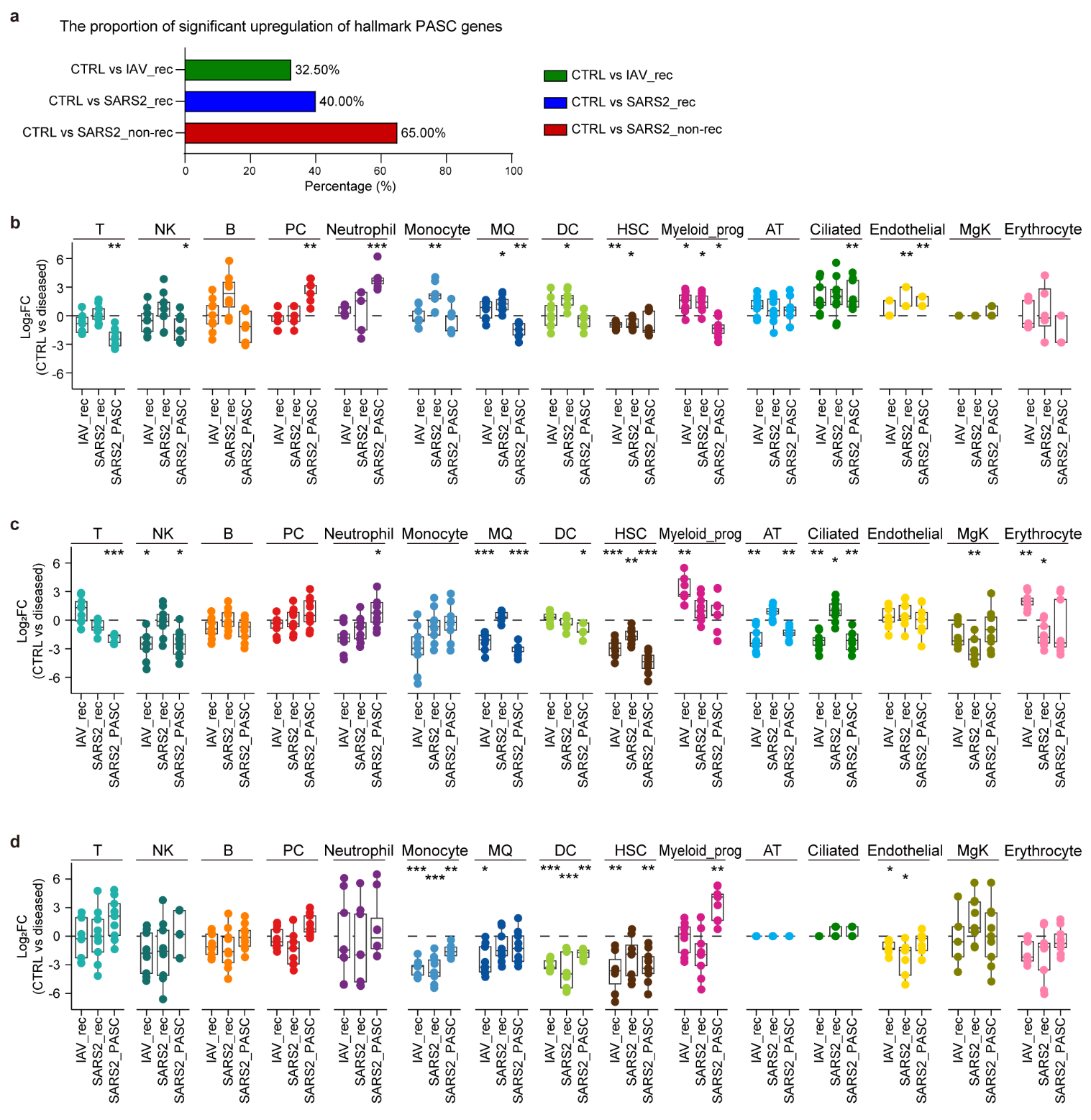


**Supplementary Fig. 2 Composition changes of cell populations for each cell type among the groups.**
**a** bar plot displaying the proportion of significantly upregulated hallmark PASC genes in diseased groups compared to controls. **b**–**d** Box plots showing the log_2_FC in cell populations for each cell type in diseased groups compared with controls, shown in BALF (**b**), lung (**c**), and spleen (**d**). The *p* values are calculated using the Wilcoxon rank-sum test. **p*< 0.05, ***p* < 0.01, ****p* < 0.001. T, T cells; NK, Natural killer cells; B, B cell; PC, Plasma cells; MQ, Macrophages; DC, Dendritic cells; HSC, Hematopoietic stem cells; Myeloid_prog, Myeloid progenitor cells; AT, Pulmonary alveolar type I and type II cells; Ciliated, Ciliated cells; Endothelial, Endothelial cells; MgK, Megakaryocytes; CTRL, control group; IAV_rec, IAV_recovery group; SARS2_rec, SARS-CoV-2_recovery group; SARS2_PASC, SARS-CoV-2_non-recovery group.


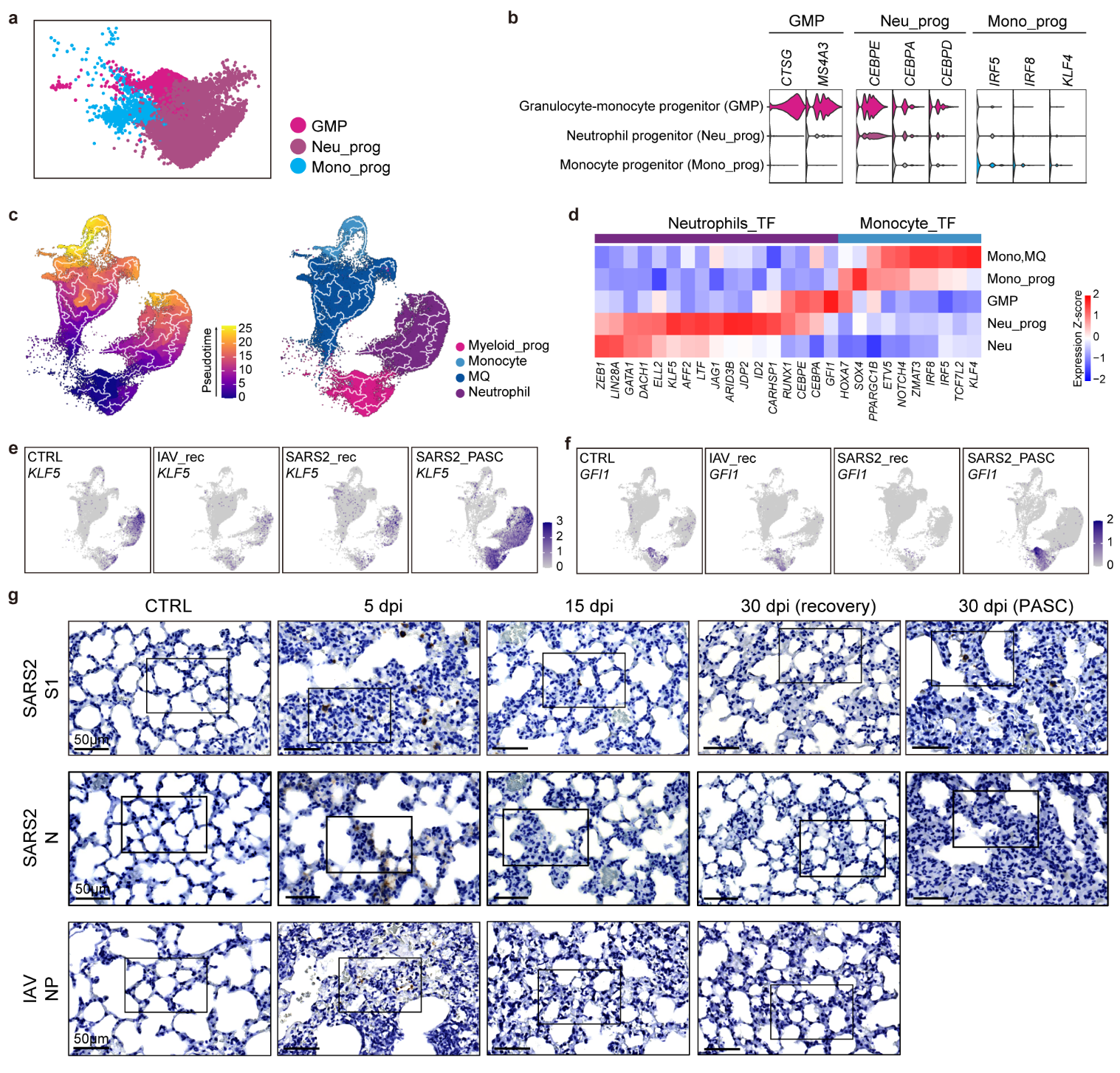


**Supplementary Fig. 3 Myeloid differentiation and viral antigen localization.**
**a** UMAP plot depicting the three distinct myeloid progenitor sub-populations: granulocyte-monocyte progenitor (GMP), neutrophil progenitor (Neu_prog), and monocyte progenitor (Mono_prog). **b** Multiple violin plots showing the expression levels of cell-type specific marker genes in the different myeloid progenitor sub-populations. **c** Two UMAP plots illustrating differentiation pathways of trajectory analyses within myeloid cells, one colored by pseudotime (left) and the other by cell type (right). **d** Heat map displaying neutrophil or monocyte transcription factor (TF) expression (represented as Z-scores per gene) at monocytes and macrophages (Mono, MQ), monocyte progenitors (Mono_prog), Granulocyte-monocyte progenitors (GMP), neutrophil progenitor cells (Neu_prog), and neutrophils (Neu) population. **e**, **f** Feature plots of *KLF5* (**e**), *GFI1* (**f**) gene expression levels per cell in myeloid populations. **g** Low-magnification images of IHC results showing viral antigens in lung tissue (scale bars, 50 μm). Each small box in images indicates the position of the high-magnification images shown in Fig. 3h. CTRL, control group; IAV_rec, IAV_recovery group; SARS2_rec, SARS-CoV-2_recovery group; SARS2_PASC, SARS-CoV-2_non-recovery group; S1, spike protein S1 subunit; N, nucleocapsid; NP, nucleoprotein.


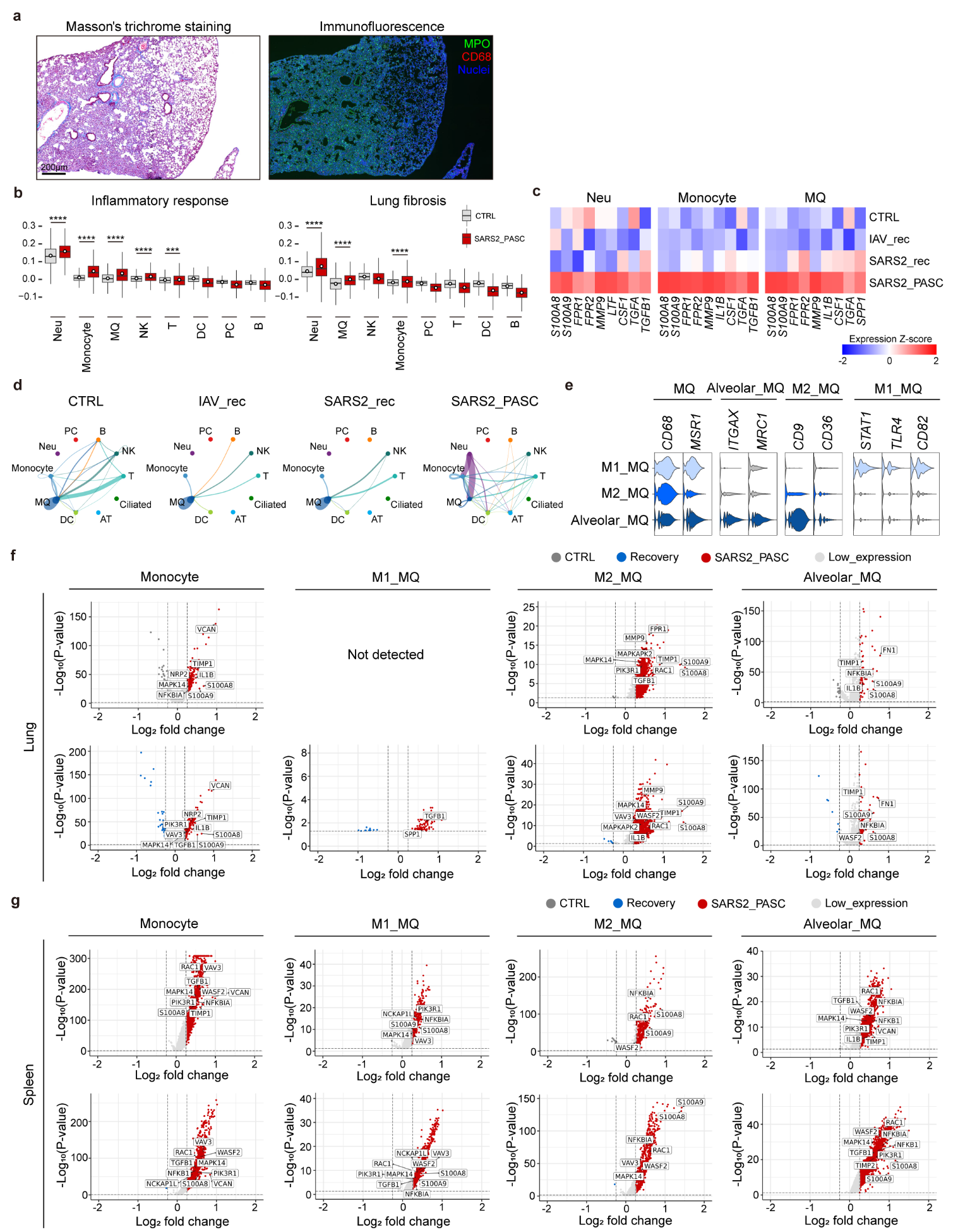


**Supplementary Fig. 4 Comparative gene expression analysis in monocyte and macrophage sub-populations**

**a** Representative MT-stained and multiplex immunofluorescence images of lung tissues showing nuclei (DAPI, blue), MPO (green), and CD68 (red) (scale bars, 200 μm). **b** Box plots showing the module scores of inflammatory response (GO:0006954) and lung fibrosis (WP3624) in immune cells from CTRL and SARS2_PASC groups. The *p* value was estimated using the Wilcoxon rank-sum test. **p* < 0.05, ***p* < 0.01, ****p* < 0.001, *****p* < 0.0001. **c** Heatmap displaying the average expression of inflammation- and fibrosis-related genes across groups in neutrophils, monocytes, and macrophages. d Circle plots illustrating TGF-β signaling interactions between immune cells and epithelial cells across groups. Edge width represents the communication probability between cell types, as inferred by CellChat. **e** Multiple violin plots illustrating the expression levels of cell-type specific marker genes in the different macrophage sub-populations. **f**, **g** Volcano plots highlighting significantly differentially expressed fibrosis-related pathway and inflammation genes in the monocyte and macrophage sub-populations in the lung (**f**) and spleen (**g**). Each panel shows log_2_FC values and *p* values between the Control (CTRL) vs SARS-CoV-2_PASC (SARS2_PASC) groups and Recovery vs SARS-CoV-2_PASC groups, using the Wilcoxon rank-sum test. Myeloid_prog, Myeloid progenitor cells; M1_MQ, M1 type macrophages; M2_MQ, M2 type macrophages; Alveolar_MQ, Alveolar macrophages.


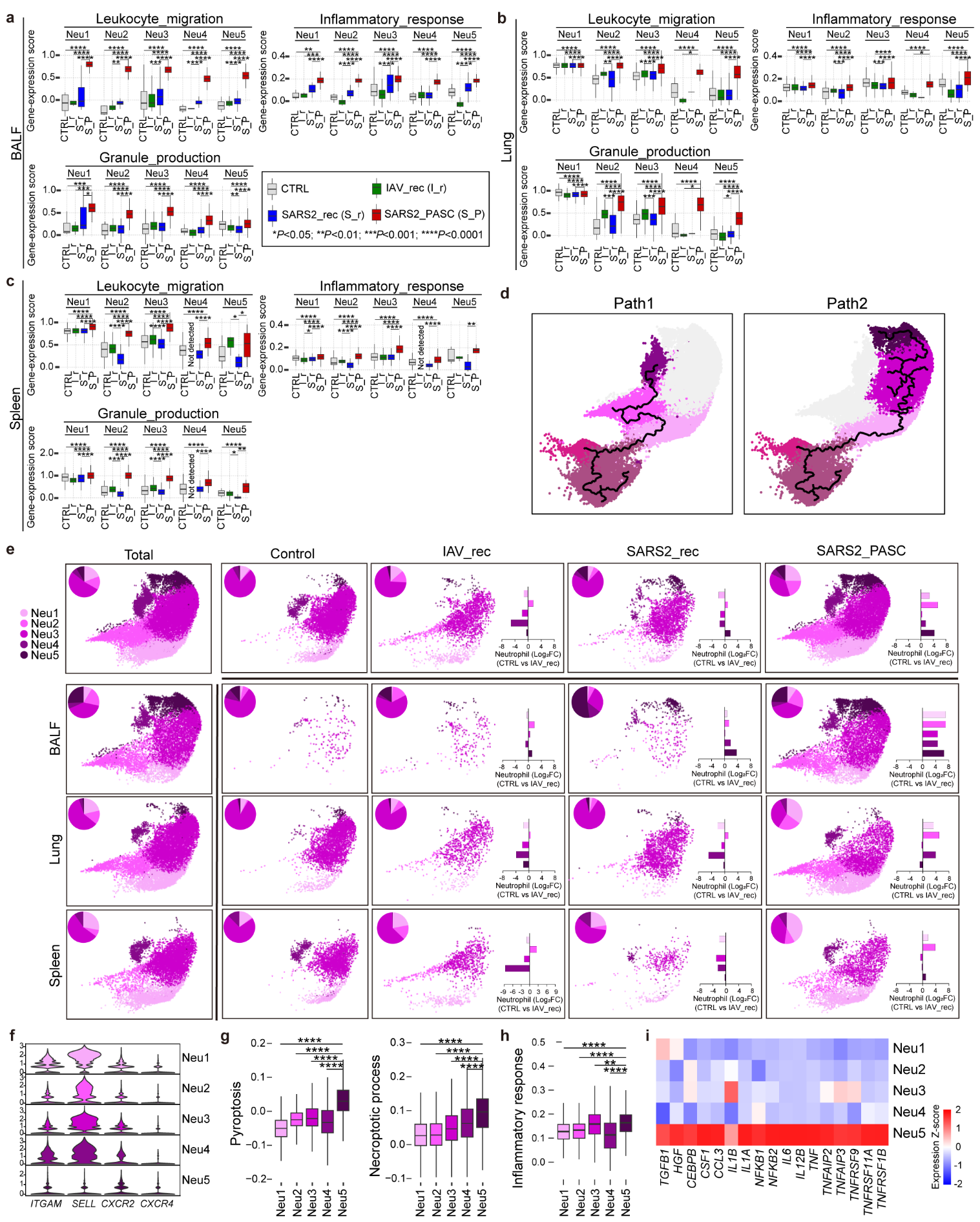


**Supplementary Fig. 5 Neutrophil function and differentiation analysis in BALF, lung, and spleen from SARS-CoV-2 infected hamsters.**
**a**-**c** Box plots displaying module scores of neutrophil functions in neutrophil sub-clusters of BALF (**a**), lung (**b**) and spleen (**c**); leukocyte migration (M12269, GO:0050900), inflammatory response (M5932), and granule production (*Evrard et al*). **d** Two separate UMAP plots depicting trajectory two-way path of differentiation of myeloid progenitor and neutrophil sub-cluster. **e** UMAPs and pie charts show the population distribution of each neutrophil sub-cluster division by all groups and tissues. In diseased groups, the neutrophil sub-clusters exhibit a relative population change compared to the control. **f** Multiple violin plot of the neutrophil maturation related gene expression in neutrophil sub-clusters. **g, h** Box plots of module scores for pyroptosis (M24370, GO:0070269) (**g**), necroptotic process (M46836, GO:0070266) (**g**), and inflammatory response (GO:0006954) (**h**) in neutrophil sub-clusters. **i** Heatmap displaying expression of lung fibrosis, NF-kB, and TNFA signaling related genes at neutrophil sub-clusters. CTRL, control group; I_r or IAV_rec, IAV_recovery group; S_r or SARS2_rec, SARS-CoV-2_recovery group; S_P or SARS2_PASC, SARS-CoV-2_non-recovery group; Neu, Neutrophil. Statistical significance was calculated by comparing all groups (**a**-**c**) or by comparing SARS2_PASC with others (**g, h**). The *p* value was estimated using the Wilcoxon rank-sum test. **p* < 0.05, ***p* < 0.01, ****p* < 0.001, *****p* < 0.0001.


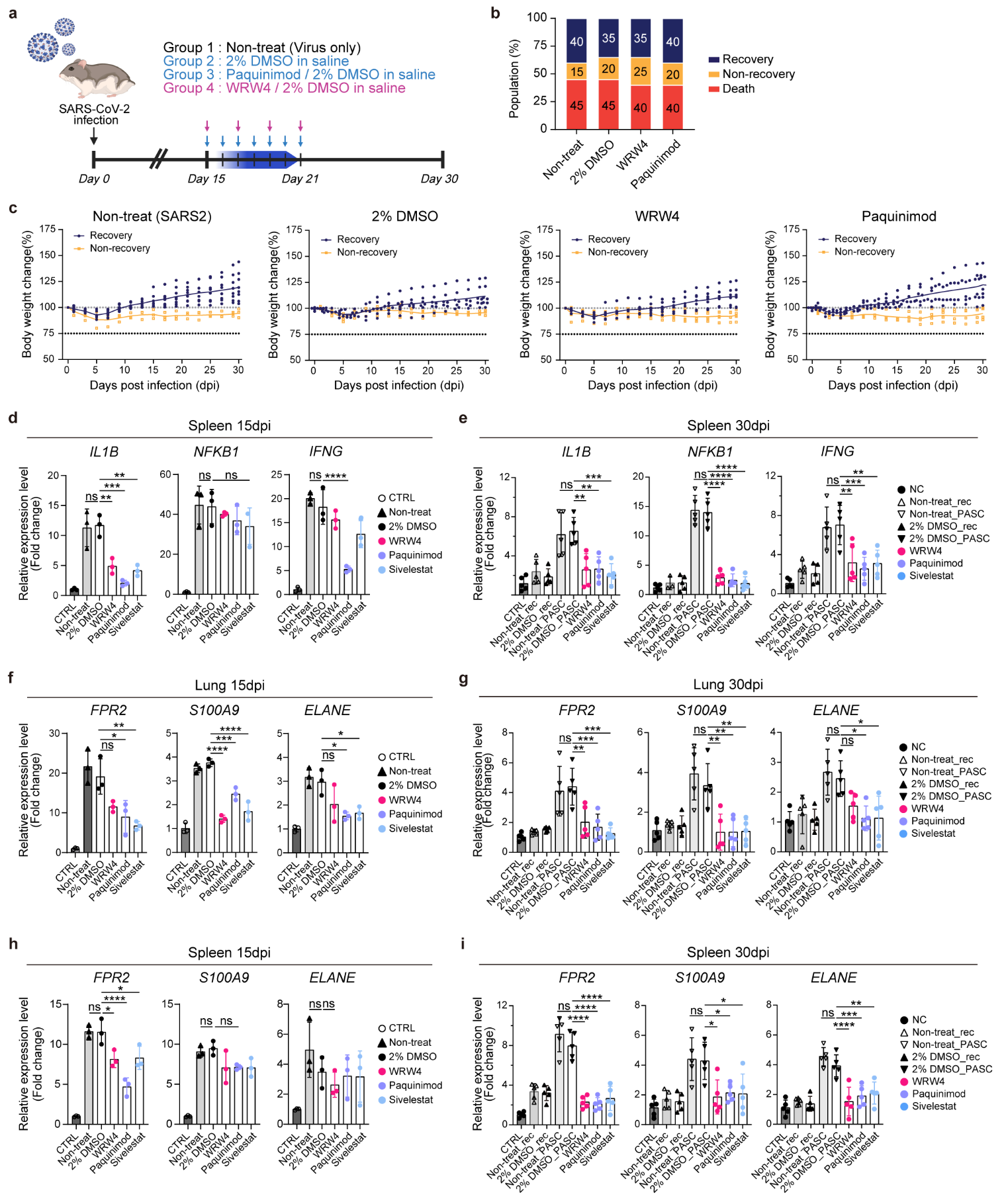


**Supplementary Fig. 6** **Early, but not delayed, drug administration reduces the expression of inflammatory cytokines and genes in lung and spleen tissues.**
**a** Schematic overview of the animal experiment. The diagram reflects each group of the inhibitor treatment schedule. Created in BioRender. Yu, M. (2025) https://BioRender.com/djv262o. **b** Proportion of recovery (blue), non-recovery (yellow), death (red) of each group. **c** Body weight change data of Non-treat (SARS2, SARS-CoV-2 infection only), 2% DMSO in a saline treat (2% DMSO), WRW4/2% DMSO in a saline treat (WRW4), and Paquinimod/2% DMSO in a saline treat (Paquinimod). **d**, **e** Relative expression levels of *IL1B, NFKB*, and *IFNG* in the spleen at 15 (**d**), and 30 (**e**) days post-infection (dpi), expressed as delta-delta Cycle threshold (Ct) values. **f**–**i** Relative expression levels of *FPR2*, *S100A9*, and *ELANE* in the lung at 15 (**f**), 30 (**g**) dpi, and in the spleen at 15 (**h**), 30 (**i**) dpi, expressed as delta-delta cycle threshold (Ct) values. Gene expressions were normalized using Glyceraldehyde 3-phosphate dehydrogenase (GAPDH) as a housekeeping gene. Data are presented as means (SD). Statistical significance is indicated as follows: **p* < 0.05, ***p* < 0.01, ****p* < 0.001, *****p* < 0.0001, ns (*p* > 0.05), one-way ANOVA. CTRL, control group; Non-treat_rec, recovery group without treatment; Non-treat_PASC, non-recovery group without treatment; 2% DMSO_rec, recovery group treated with 2% DMSO in saline; 2% DMSO_PASC, non-recovery group treated with 2% DMSO in saline.

**
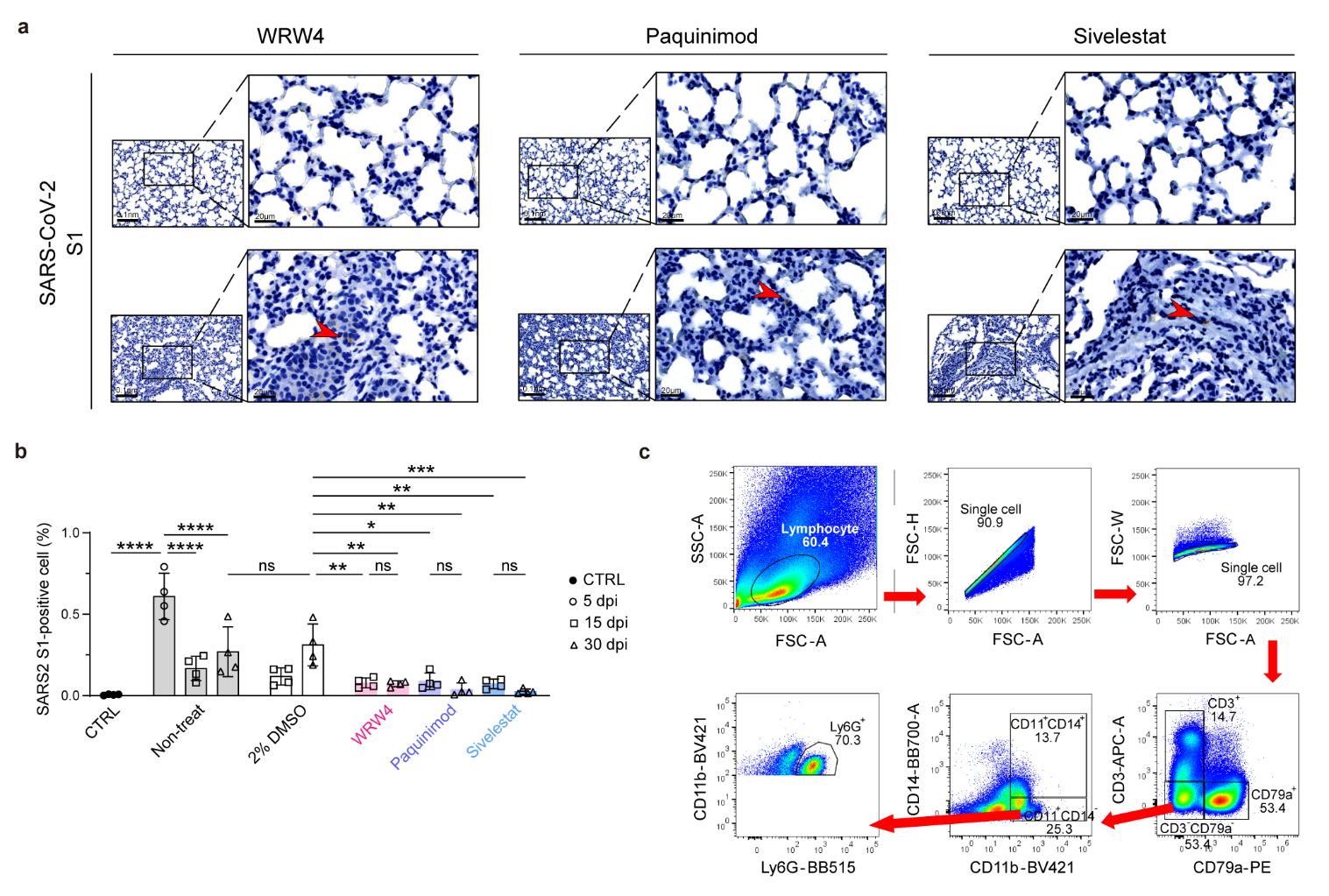
**

**Supplementary Fig. 7 Reduced presence of S1 antigen and immune cell gating strategy following drug treatment.**

**a** Representative immunohistochemistry (IHC) images showing SARS-CoV-2 Spike S1 antigen in lung tissues of three drug-treated groups (WRW4, Paquinimod, and Sivelestat) at 30 dpi. Antigen-positive cells are indicated by red arrowheads (scale bars, 20㎛). **b** Quantification of the percentage of SARS-CoV-2 (SARS2) S1-positive cells in lung tissue from different groups: control (CTRL), Non-treat (SARS2, SARS-CoV-2 infection only), 2% DMSO in a saline (2% DMSO), WRW4 in 2% DMSO (WRW4), Paquinimod in 2% DMSO (Paquinimod), and Sivelestat in 2% DMSO (Sivelestat) at different dpi. Data are presented as means (SD). **c** Flow cytometry gating strategy for analyzing B cells (CD79⁺), T cells (CD3⁺), monocytes/macrophages (MQs) (CD11b⁺CD14⁺), and neutrophils (CD11b⁺Ly6G⁺)**.** Statistical significance is indicated as follows: **p* < 0.05, ***p* < 0.01, ****p* < 0.001, *****p* < 0.0001, ns (*p* > 0.05), one-way ANOVA.

**Supplementary Tables**

**Supplementary Table 1.** List of primer sequences. Related to Materials and Methods.

| Gene | | 5' to 3' |
| --- | --- | --- |
| SARS-CoV-2 E gene | Forward | ATG TAC TCA TTC GTT TCG GAA GAG |
| SARS-CoV-2 E gene | Reverse | TTA GAC CAG AAG ATC AGG AAC TCT AG |
| IAV M gene | Forward | ATG AGC CTT TTA ACC GAG GTC GAA ACG |
| IAV M gene | Reverse | TGG ACA AAT CGT CTA CGC TGC AG |
| *P. roborovskii* hamster (PHOROB)_GAPDH | Forward | AAC TAC ATG GTC TAT ATG TTC CAG TAT GAC |
| PHOROB_GAPDH | Reverse | AGA CAC CAG TAG ATT CCA CAA CAT AC |
| PHOROB_FPR2 | Forward | ATG TGT AAA TTA GTT CAC ATT GTG GTG G |
| PHOROB_FPR2 | Reverse | TGG GCA ATG TGA GGA TGA GAG |
| PHOROB_S100A9 | Forward | CTG TTG GAG CGC AGC ATA A |
| PHOROB_S100A9 | Reverse | CTT CAG AAA GTT TGC CAG ATC AG |
| PHOROB_ELANE | Forward | TCA TCG CCA GGA ACT TCG T |
| PHOROB_ELANE | Reverse | GTG GCT GAG CCA TTG AGC |
| PHOROB_IL1B | Forward | TTC GAC AGC TGC ACT GC |
| PHOROB_IL1B | Reverse | CAC ACA GGA CAG GTA CAG ATT CT |
| PHOROB_IFNG | Forward | GTT GCT CTG CCT CAC CCA |
| PHOROB_IFNG | Reverse | AAA AGG AGA CAA TCT GGC TCT CA |
| PHOROB_NFKB | Forward | TGC AGC AGA CGA AGG AGA |
| PHOROB_NFKB | Reverse | CGG TTA CAC ACC CTG CAG |

**Supplementary Table 2.** Quality of single-cell RNA sequencing (scRNA-seq) data. Related to Fig. 2

| **Tissue** | **Condition** | **Replicate** | **Number of cells** | **Median gene per cell** | **Median UMI per cell** | **Mean MT percent** |
| --- | --- | --- | --- | --- | --- | --- |
| BALF | CTRL | 1 | 1,615 | 3,813 | 15,649 | 2.3634 |
| BALF | CTRL | 2 | 3,019 | 3,882 | 17,493 | 2.6502 |
| BALF | CTRL | 3 | 1,806 | 3,178 | 11,509 | 2.7235 |
| BALF | IAV_rec | 1 | 1,616 | 2,376 | 7,889 | 1.9740 |
| BALF | IAV-rec | 2 | 4,442 | 2,346 | 8,511 | 1.6382 |
| BALF | IAV-rec | 3 | 3,784 | 2,187 | 7,750 | 1.1964 |
| BALF | SARS2_rec | 1 | 8,007 | 1,490 | 2,856 | 3.1335 |
| BALF | SARS2_rec | 2 | 6,743 | 2,208 | 5,997 | 2.1506 |
| BALF | SARS2_rec | 3 | 4,277 | 4,199 | 16,040 | 2.5567 |
| BALF | SARS2_PASC | 1 | 8,010 | 613 | 1,838 | 1.1807 |
| BALF | SARS2_PASC | 2 | 1,301 | 1,575 | 6,571 | 1.3772 |
| BALF | SARS2_PASC | 3 | 1,572 | 2,524 | 11,070 | 2.3567 |
| lung | CTRL | 1 | 3,089 | 2,179 | 6,148 | 3.5234 |
| lung | CTRL | 2 | 7,713 | 1,149 | 2,393 | 3.5614 |
| lung | CTRL | 3 | 4,882 | 1,607 | 4,411 | 3.3263 |
| lung | IAV_rec | 1 | 3,184 | 891 | 1,629 | 1.4088 |
| lung | IAV-rec | 2 | 2,566 | 863 | 2,207 | 1.9103 |
| lung | IAV-rec | 3 | 8,514 | 1,298 | 2,874 | 3.2910 |
| lung | SARS2_rec | 1 | 2,834 | 745 | 1,397 | 2.9934 |
| lung | SARS2_rec | 2 | 5,542 | 1,234 | 2,694 | 2.8174 |
| lung | SARS2_rec | 3 | 5,677 | 1,534 | 3,734 | 2.4018 |
| lung | SARS2_PASC | 1 | 6,852 | 1,434 | 5,028 | 1.8581 |
| lung | SARS2_PASC | 2 | 3,090 | 587 | 1,131 | 1.2471 |
| lung | SARS2_PASC | 3 | 1,778 | 2,383 | 11,917 | 1.7369 |
| spleen | CTRL | 1 | 10,466 | 1,201 | 2,158 | 6.4477 |
| spleen | CTRL | 2 | 4,332 | 721 | 1,424 | 0.0828 |
| spleen | CTRL | 3 | 6,324 | 1,271 | 3,555 | 5.8946 |
| spleen | IAV_rec | 1 | 1,583 | 725 | 1,287 | 1.5732 |
| spleen | IAV_rec | 2 | 1,785 | 1,050 | 3,052 | 1.3547 |
| spleen | IAV_rec | 3 | 2,476 | 722 | 1,483 | 2.3012 |
| spleen | SARS2_rec | 1 | 855 | 762 | 1,181 | 0.4104 |
| spleen | SARS2_rec | 2 | 1,395 | 1,260 | 2,001 | 0.3239 |
| spleen | SARS2_rec | 3 | 6,049 | 1,295 | 2,218 | 4.8082 |
| spleen | SARS2_PASC | 1 | 8,227 | 1,590 | 4,721 | 3.2064 |
| spleen | SARS2_PASC | 2 | 6,538 | 2,168 | 6,300 | 2.9876 |
| spleen | SARS2_PASC | 3 | 6,173 | 2,741 | 11,974 | 2.3652 |
|  |  |  | 158,116 (sum) | 1,462 (median) | 3,644 (median) | 2.4204 (mean) |
